# Dynamic Analysis of Stochastic Transcription Cycles

**DOI:** 10.1371/journal.pbio.1000607

**Published:** 2011-04-12

**Authors:** Claire V. Harper, Bärbel Finkenstädt, Dan J. Woodcock, Sönke Friedrichsen, Sabrina Semprini, Louise Ashall, David G. Spiller, John J. Mullins, David A. Rand, Julian R. E. Davis, Michael R. H. White

**Affiliations:** 1 Centre for Cell Imaging, School of Biological Sciences, University of Liverpool, Liverpool, United Kingdom; 2 Department of Statistics, University of Warwick, Coventry, United Kingdom; 3 Warwick Systems Biology Centre, University of Warwick, United Kingdom; 4 Endocrinology Group, School of Biomedicine, University of Manchester, Manchester, United Kingdom; 5 Queen's Medical Research Institute, University of Edinburgh, Edinburgh, United Kingdom; Johns Hopkins University, United States of America

## Abstract

In individual mammalian cells the expression of some genes such as prolactin is highly variable over time and has been suggested to occur in stochastic pulses. To investigate the origins of this behavior and to understand its functional relevance, we quantitatively analyzed this variability using new mathematical tools that allowed us to reconstruct dynamic transcription rates of different reporter genes controlled by identical promoters in the same living cell. Quantitative microscopic analysis of two reporter genes, firefly luciferase and destabilized EGFP, was used to analyze the dynamics of prolactin promoter-directed gene expression in living individual clonal and primary pituitary cells over periods of up to 25 h. We quantified the time-dependence and cyclicity of the transcription pulses and estimated the length and variation of active and inactive transcription phases. We showed an average cycle period of approximately 11 h and demonstrated that while the measured time distribution of active phases agreed with commonly accepted models of transcription, the inactive phases were differently distributed and showed strong memory, with a refractory period of transcriptional inactivation close to 3 h. Cycles in transcription occurred at two distinct prolactin-promoter controlled reporter genes in the same individual clonal or primary cells. However, the timing of the cycles was independent and out-of-phase. For the first time, we have analyzed transcription dynamics from two equivalent loci in real-time in single cells. In unstimulated conditions, cells showed independent transcription dynamics at each locus. A key result from these analyses was the evidence for a minimum refractory period in the inactive-phase of transcription. The response to acute signals and the result of manipulation of histone acetylation was consistent with the hypothesis that this refractory period corresponded to a phase of chromatin remodeling which significantly increased the cyclicity. Stochastically timed bursts of transcription in an apparently random subset of cells in a tissue may thus produce an overall coordinated but heterogeneous phenotype capable of acute responses to stimuli.

## Abstract 2

Cycling of gene expression in individual cells is controlled by dynamic chromatin remodeling.

## Author Summary

Timing of biological processes such as gene transcription is crucial to ensure that cells and tissues respond appropriately to their environment. Until recently it was assumed that most cells in a tissue responded in a similar way, and that changes in cellular activity were relatively stable. However, studies of messenger RNA and protein levels in single cells have shown the presence of considerable heterogeneity. This suggested that transcription in single cells may be highly dynamic over time. Using a combined experimental and theoretical approach, with time-lapse imaging of reporter gene expression over 25 h periods, we measured the rate of prolactin gene transcription in single pituitary cells and detected clear cycles of transcriptional activity. Mathematical analysis, using a binary model that assumed transcription was on or off, showed that these cycles were characterized by a minimum refractory period that involved chromatin remodeling. The timing of transcription from two different reporter constructs driven by identical promoters in the same cell was out-of-phase, suggesting that the pulses of gene expression are due to processes intrinsic to expression of a particular gene and not to environmental effects. We further show that the pulses of transcription are independent chromatin cycles at each gene locus. Therefore, heterogeneous patterns of gene expression may facilitate flexible transcriptional responses in cells within intact tissue, while maintaining a well-regulated average level of gene expression in the resting state.

## Introduction

Gene expression in living cells is dynamic and unstable, and fluctuations in transcription may be subject to stochastic regulation of processes including transcription factor and polymerase recruitment, and chromatin remodeling [1]–[5]. Cell-to-cell variation in the amount of protein a gene encodes is generally thought to arise from the typically small number of molecules (e.g. gene copies), which are involved in gene expression. The factors leading to this variation have been defined by studies in prokaryotes and lower eukaryotes as either *extrinsic* (deriving from variations in global, cellular factors, such as varying amounts of transcriptional activators) or *intrinsic* (i.e. inherently random molecular events, such as the transcription of mRNA or translation of proteins) [4],[6],[7]. Previous studies addressing the characterization of intrinsic and extrinsic noise have mainly focused on bacteria and yeast models, often using pairs of reporter genes to assess heterogeneity in protein levels as an indirect readout of expression level at a fixed time-point [4],[6]. One study has reported a similar fixed time-point analysis in single human cells using dual fluorescent protein read-out [7]. Short-term transcriptional pulses (bursts) have been observed in both prokaryotes [2],[3],[8] and eukaryotes [5],[6],[9],[10]. Chromatin remodeling has been suggested as one possible intrinsic source of variation that may lead to the intense stochastic transcriptional bursts that have been shown to occur in eukaryotic gene expression [5],[6],[10].

In mammalian cells, the variation in transcription between cells has been most quantitatively studied by using fluorescent in situ hybridization analysis [10]–[12]. However, these studies do not provide long-term time-course analysis in single cells. One approach to provide real-time semi-quantitative analysis of transcription is the imaging of reporter gene expression—for example, using firefly luciferase [13]–[15]. Such studies support the view that gene expression is very dynamic over long time periods and occurs in transcriptional bursts of varying duration that are not coordinated between different cells. To date, the key aim of understanding real-time dynamics by directly quantifying transcription rates of multiple genes over time in single cells has not been achieved.

One gene that displays dynamic transcription and marked heterogeneity between cells is prolactin (PRL) [14],[16]–[18]. Prolactin is a hormone secreted by pituitary lactotrophic cells that is important for reproductive function, lactation, and control of fertility. Pituitary tumors secreting prolactin are common in man, and the hormonal regulation of prolactin secretion and gene expression has therefore been extensively studied [19]–[22]. In the present study, we used single cell reporter gene imaging to explore the pulsatile and cyclical nature of the transcription of PRL. Using measurements of mRNA and protein stability, we were able to quantify transcription rates from separately integrated reporter genes within the same cells and compare the kinetics of transcription over time. Analysis of the response to acute signals and the manipulation of histone acetylation suggested that dynamic chromatin changes control cycle timing.

## Results

### Cycles in Prolactin Transcription in Single Pituitary Cells

**Figure 1:**
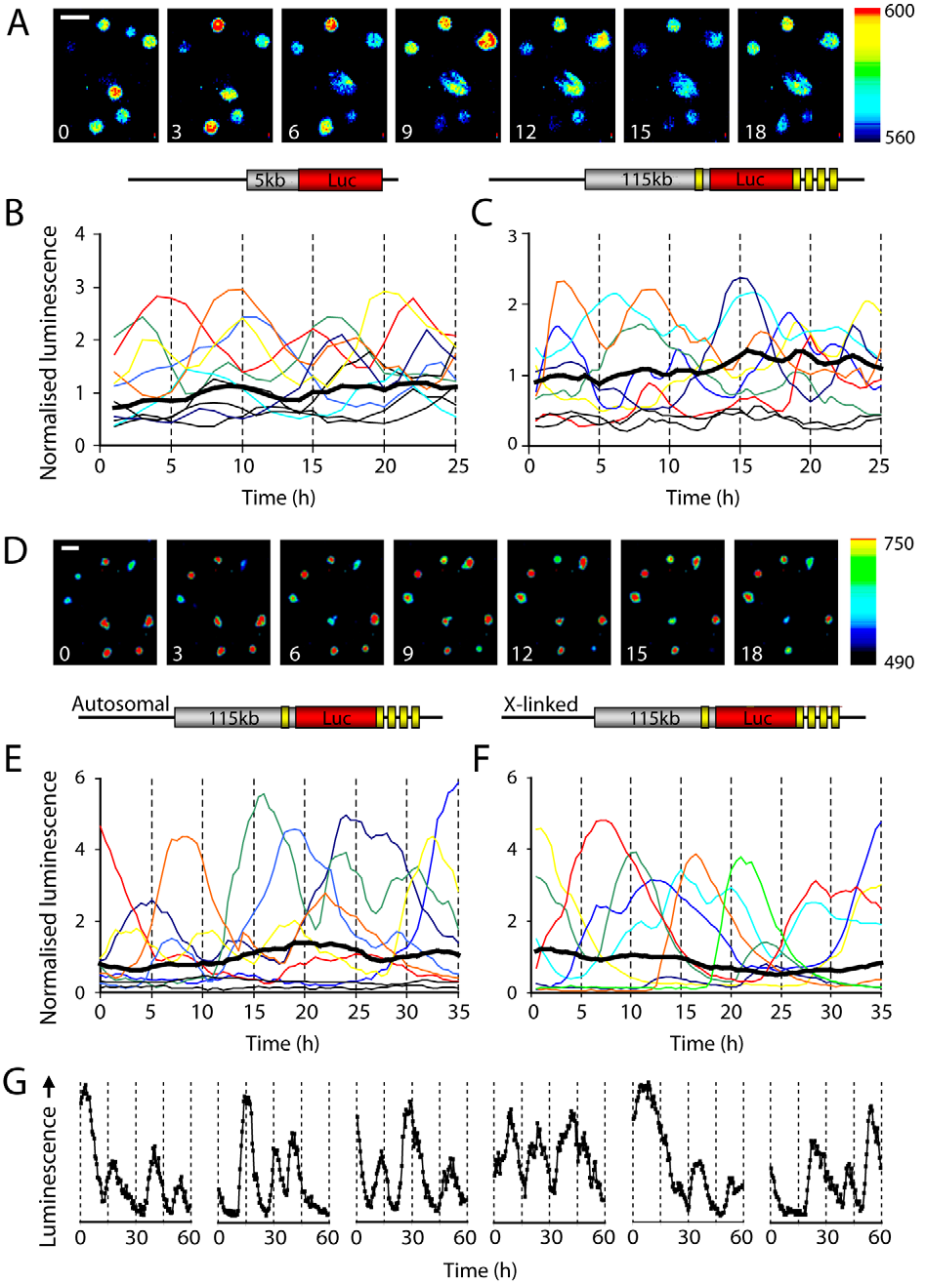
Heterogeneous transcription cycles in single living cells. Luminescence signal from (A and B) the rat pituitary GH3 cell line stably transfected with a luciferase reporter gene under the control of the *hPRL* 5,000 bp exon 1b promoter (GH3-DP1 cells), (C) GH3 cells stably expressing luciferase under the control of a 160 kb *hPRL* genomic fragment (*hPRL-Luc* BAC), (D–F) primary cultures of pituitary cells from transgenic rats expressing luciferase under the control of the *hPRL-Luc* BAC with (D and E) autosomal, and (F) X-linked transgene insertion sites. The colored lines represent data from single cells, and the average population response is shown in each graph by a thick black line (B, *n* = 15 cells; C, *n* = 18 cells; E, *n* = 22 cells; F, *n* = 20 cells). (G) Traces from individual transgenic primary cells over extended time periods. Numbers in each image series represent time in hours. Bars in image series represent 50 µm. Different regions of the promoter-reporter genes are represented in the schematic diagram by 5′- or 3′-flanking regions (grey), luciferase reporter sequence (red), and *hPRL* exons 1a and 2–5 (yellow, not to scale).

Human PRL (hPRL) promoter-directed transcription was heterogeneous and dynamic in rat pituitary GH3 cells using luciferase reporter genes. Transcriptional pulses were observed in GH3 cell lines stably expressing a 5 kb *hPRL-Luciferase* (*hPRL-Luc*) reporter gene [14] or a larger 160 kb *hPRL* genomic locus reporter, a *hPRL*-*Luc* Bacterial Artificial Chromosome (BAC, [16]; Figure 1A, B, and C; Figure S1). Similar patterns were observed in primary cultures of pituitary cells (taken from transgenic rats [16]), where the *hPRL*-*Luc* BAC was integrated either into an autosomal (Figure 1D and E) or an X-chromosome locus (Figure 1F). In all four model systems distinct transcriptional cycles were discerned (e.g. Figure 1G) showing that these responses were not affected by promoter length or integration site.

### Uncorrelated Transcription Cycles from Two Identical Promoters in a Single Cell

**Figure 2:**
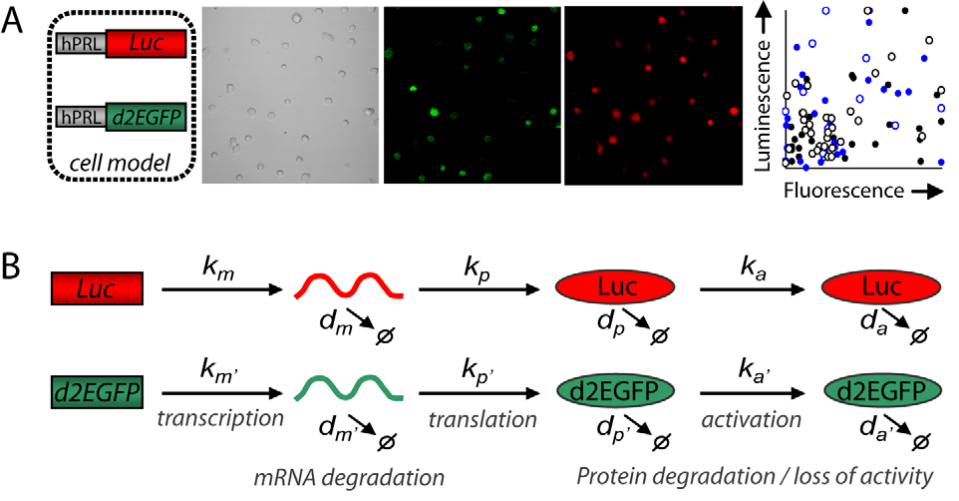
Measurement of transcription from two reporter genes driven by identical promoters in the same single cell. (A) Luminescence and fluorescence measurements from GH3 cells stably expressing both the *hPRL-Luc* and *hPRL-d2EGFP* reporter genes. Intensity of the luminescence and fluorescence signal from single cells fails to correlate (data from 96 cells, four experiments are shown, depicted by different symbols, *r* ^2^ = 0.09). (B) A schematic diagram showing the conversion of transcription rate from *hPRL-Luc* and *hPRL-d2EGFP* reporter gene data. Images from each reporter gene at a single time-point are shown, as are the model parameters required to convert luciferase and d2EGFP reporter protein data into transcription rate.

Pulses in gene expression in individual cells could arise from the transcription process itself or from signaling events reflecting the cellular environment. To discriminate between these possibilities, a dual-transgene cell line was constructed expressing separate luciferase and d2EGFP (destabilized enhanced Green Fluorescent Protein) reporter genes under the control of identical 5 kb *hPRL* promoters, integrated as independent gene copies (GH3-DP1 cell line; Figure 2A; one or at most two copies; Figure S2, Figure S3). The luciferase and d2EGFP reporter genes were selected due to their reported short protein half-lives. The use of these very different reporter genes (which have different chemistries for formation of the signal) was considered an advantage because we could measure them entirely independently.

Signal was detected from both reporter genes, but the intensity of the expression of the reporter genes within single cells failed to correlate when measured at a single time-point (Figure 2A, Figure S4). To measure the profiles of expression from each reporter gene, fluorescence and luminescence intensities were captured from the same field of single cells over several hours (Figure S5). Due to the different mRNA and protein half-lives of these two reporter genes (Figure S2, Figure S10, Table S1, Text S1 Section 3) direct comparison between the timing of expression could not be made. Therefore, in order to make quantitative comparisons between the timing of expression of these two different reporter genes within the same single cell, a mathematical model was developed (Figure 2B, [23]) which used statistical analysis to reconstruct estimates of the time-dependent transcription rate from the reporter imaging data (Figure S11, Figure S14, Text S1 Section 3).

**Figure 3:**
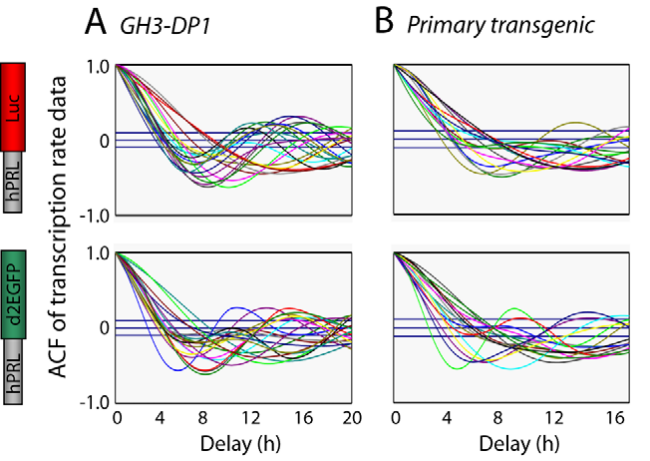
Cycles of prolactin transcription from separate reporter genes within a single cell. (A and B) Autocorrelation analysis of the reconstructed transcription rate dynamics from the *hPRL-Luc* and *hPRL-d2EGFP* reporter genes within the same single cells. (A) GH3-DP1 cells (*n* = 20 cells) and (B) primary transgenic cells (*n* = 16 cells) expressing the *hPRL-Luc* and *hPRL-d2EGFP* BAC genes.

Autocorrelation analysis was performed on the reconstructed transcription rates from the *hPRL-Luc* and the *hPRL-d2EGFP* reporter genes in the dual-reporter GH3-DP1 cell line. This showed that transcription cycles were occurring at each gene with a dominant period of 11.3±3.3 h (Figure 3A and Figure S9). This value was measured from the dual reporter experiments and possibly provided an underestimate due to the limited timeframe of the experiments. Cycles of *hPRL* transcription were also observed from both luciferase and d2EGFP reporter genes in individual clonal pituitary cells from dual BAC-reporter transgenic rats grown in primary culture (see Materials and Methods, [16]), with a slightly longer period (15.2±4.8 h; Figures 3B, S1, and S8).

**Figure 4:**
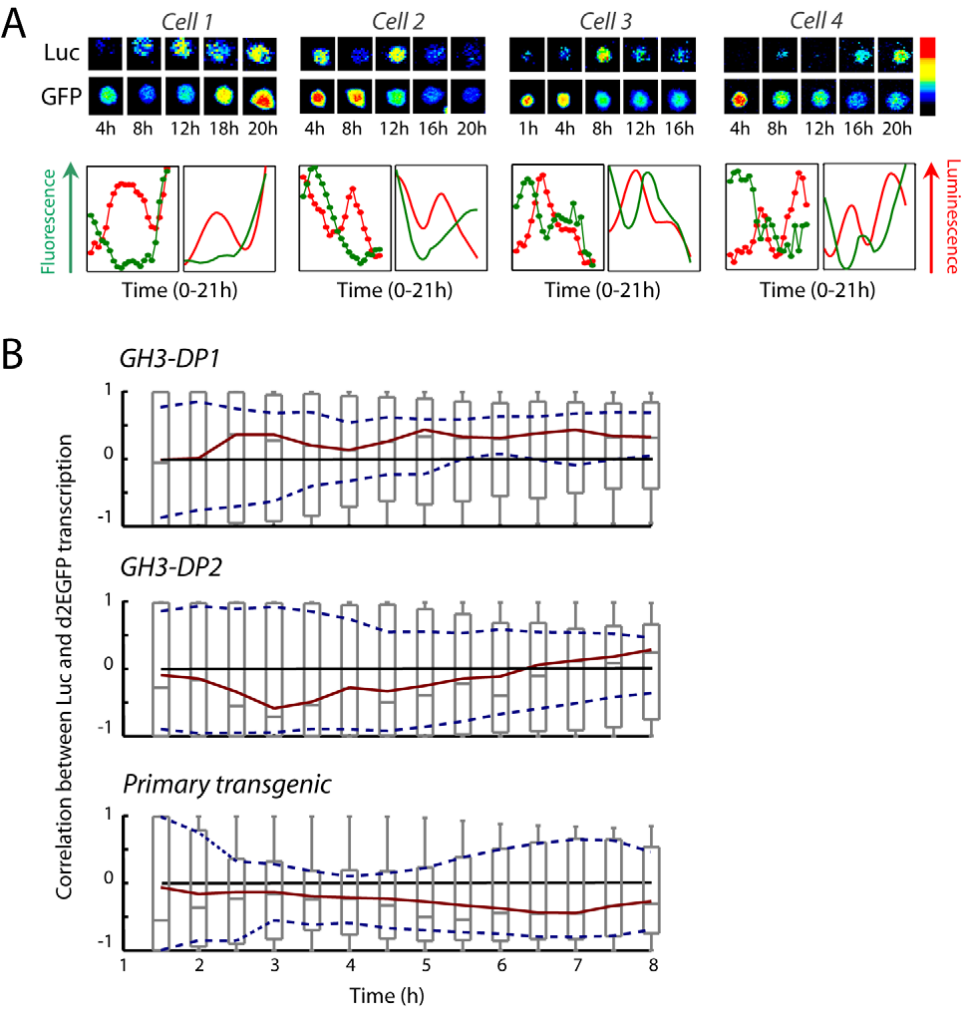
Uncorrelated cycles of gene expression from dual reporter genes in single cells. (A) Comparison of the dynamics of *hPRL-Luc* and *hPRL-d2EGFP* in four representative single cells. Top panels show luminescence and fluorescence images for each cell, and graphs show the dynamics of the two reporter genes from the same single cell over time (left-hand graphs, reporter-gene profiles; right-hand graphs, reconstructed transcription rates). (B) Lack of correlation over time between the transcription rates for two identical *hPRL* promoters in unstimulated conditions in two independent single cell clones (GH3-DP1, *n* = 83 cells; GH3-DP2, *n* = 36 cells) and primary transgenic pituitary cells (primary, *n* = 22 cells). The sequence of boxplots against time (T, *x*-axis) shows the distribution of the correlation coefficients between the timing of transcription from the two reporter genes over the cells within each pooled group (over rising increments from 1.5 h to 8 h). The red lines indicate median and the dotted blue lines show the 95% confidence interval for the median. If the zero line occurs within dotted lines, then the median is not significantly different from zero.

The ability to obtain quantitative data for the time-dependent transcription rates from the two reporter genes enabled us to ask whether the transcription cycles observed at individual loci were temporally coordinated or were out-of-phase within a single cell (Figure 4A, Figure S6). We analyzed the rank correlation coefficient *C(T)* between the transcription time-series for the two reporter genes over a time window of length *T* for increasing values of *T* (see Text S1 Section 3.3). In unstimulated conditions there was no significant correlation (*p*<.05) in the timing of transcription cycles between the dual reporters in the same single cell (Figure 4A and B). In order to show that this was not an artifact of the genomic integration site we investigated independently derived cell lines: no significant correlation between the two promoters was detected in two different clones of the stably transfected cell lines or in dual-reporter transgenic primary cells (Figure 4B, Figure S12). These data demonstrate that cycles of *hPRL*-promoter activity did not depend on promoter length or on transgene integration site. Most importantly, the lack of correlation between the timing of *hPRL* transcription from promoters within the same cell in time-lapse imaging experiments showed that the expression cycles from distinct loci in a single cell were not synchronized or temporally coordinated. The fact that the cycles at individual loci in unstimulated single cells were uncorrelated (and that this phenomenon occurred in both cell lines and post-mitotic primary pituitary cells) suggested that the pulses in PRL gene expression were independent of cell cycle stage. This is in agreement with our previous study that suggested that variation in hPRL-luciferase reporter expression was independent of cell cycle in the GH3 cells (which have a cell cycle timing of approximately 40 h) [18]. Overall, these data therefore suggest that the transcriptional pulses were not a reflection of the cellular status, environment, or autocrine cell signaling but rather were due to an intrinsic property of the transcription process itself.

### Evidence of a Refractory Phase between Transcription Cycles

**Figure 5:**
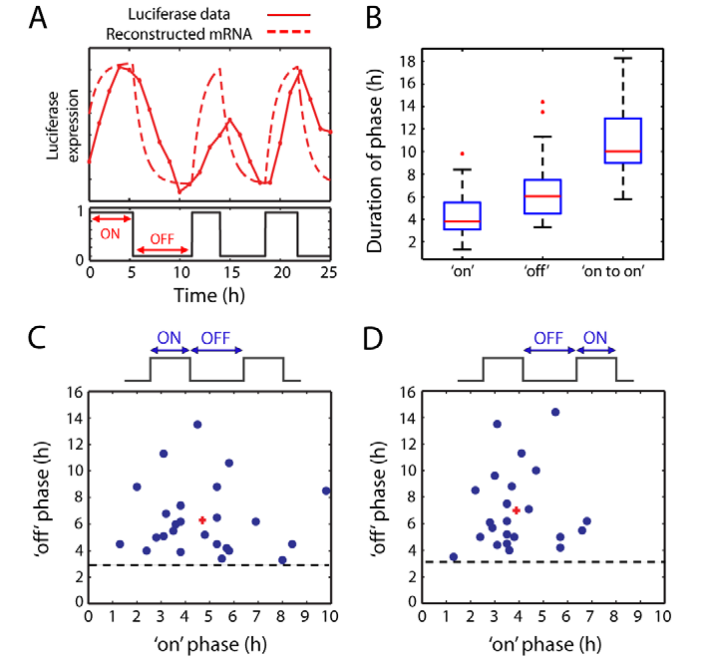
A binary model of transcription reveals transcription burst dynamics. (A) Transcription “on” and “off” times were estimated using a stochastic binary (switch) model from *hPRL-Luc* reporter gene data from GH3-DP1 cells. (B) Estimates of transcription on duration, transcription off duration, and cycle period (on to on) were calculated for each cell (*n* = 35 cells) and the results given as boxplots. The red line indicates the median of the estimates, the blue box contains values lying between the lower quartile (shortest 25%) and upper quartile (longest 25%) of the estimates, and the black lines show the range of duration estimates up to the adjacent values. Outliers are shown as red crosses. (C) A scatter plot showing the relationship between the on duration and subsequent off durations within a single cell, and (D) vice versa. The minimum off period is indicated with a dotted line in (C) and (D), and the median is displayed as a red cross.

In order to further understand and quantify the dynamics of transcriptional switching between “on-” and “off-”phases, we developed a stochastic binary switch model of transcriptional timing and used statistical algorithms to assess the distribution of times of transcriptional switching between the on and off states (Figure 5A, Text S1 Section 3). Transcription of the mRNA and translation and activation of the corresponding protein were modeled using a stochastic differential equation with binary on-off transcription and were fitted to time-series imaging data using a Markov Chain Monte Carlo (MCMC) algorithm (Figure 5A). This produced relatively tight distributions on the levels and the timing of transcription in the on and off periods (Text S1 Section 3.4, example in Figures S15–S17). This model was used to estimate the average and distribution of the times of luciferase transcriptional switching in the GH3-DP1 stable cell line and gave a dominant overall cycle period of 11.0±3.3 h (Figure 5B), which was in close agreement with the independent autocorrelation analysis described above (11.3 h; Figure 3B). Furthermore, we estimated that there was an average on-phase duration of 4.0±1 h (which was slightly longer than the timing previously described for transcriptional bursts in mammalian cells [24]). The average off-phase duration was 6.5±2 h per transcription cycle. No relationship was detected between the duration of the transcription on-phase and the preceding or subsequent off-phase (Figure 5C and D).

There was strong evidence for a refractory period of approximately 3 h, in which cells cannot respond to a stimulus with a further transcriptional pulse. For each cell studied, the mean length of the off periods never fell below 3 h (Figure 5C and D). Thus, two distinct types of mathematical analyses indicated a similar duration for transcriptional cycles, and the stochastic binary switch model further suggested that the transcriptional on- and off-phases were independent, with each having defined average and minimum durations that may account for the kinetics of these cycles.

### The Role of Signaling and Chromatin in the Transcriptional Cycles

**Figure 6:**
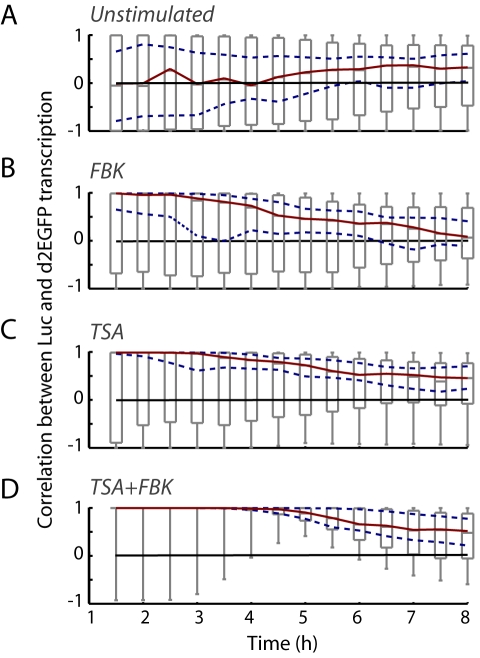
The effect of stimulation on the correlation of prolactin transcription cycles from different reporter genes. The correlation between transcription rate profiles from the two identical *hPRL* promoters in (A) unstimulated GH3-DP1 cells or following stimulus with (B) FBK, (C) TSA, or (D) combined TSA+FBK. The sequence of boxplots against time is shown (as in Figure 4B; unstimulated, *n* = 119 cells; FBK, *n* = 87 cells; TSA, *n* = 74 cells; TSA+FBK, *n* = 41 cells). Greater correlation is observed between reporter genes following stimulation with TSA+FBK.

**Figure 7:**
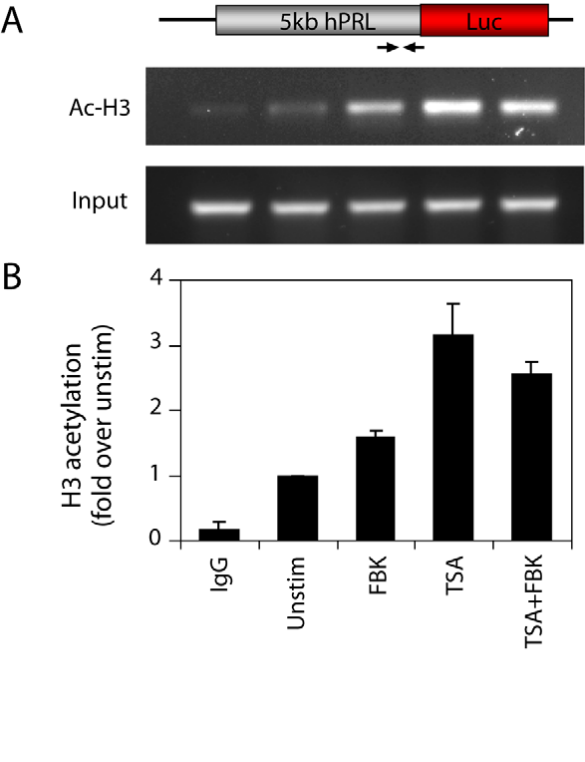
Evidence of chromatin modification in the regulation of prolactin transcription cycles. (A) Chromatin immunoprecipitation analysis of acetylated histone H3 DNA binding at the *hPRL* promoter in unstimulated conditions and following 2 h treatment with FBK, TSA, or TSA+FBK. (B) Densitometric analysis from two independent experiments with intensity normalized to unstimulated conditions (mean±SD).

The regulation of the transcriptional cycles from the *hPRL* promoter was then investigated by exposing GH3-DP1 cells to (1) combined forskolin and BayK-8644 (FBK) to activate both cAMP and Ca^2+^ signaling (Figure S7, [25]), (2) Trichostatin A (TSA, a histone deacetylase inhibitor), or (3) both treatments combined (TSA+FBK) (Table S2). All three experimental treatments resulted in an initial synchronization between the transcription profiles of the two independent *hPRL* promoter-reporter transgenes: correlation between the profiles of the dual reporters was initially very high (close to 1), both between the two transgenes within individual cells (Figure 6) and between different cells (Figure S13). This period of high correlation lasted longer following TSA treatment (Figure 6C), when compared to the very transient synchronizing effect of FBK (Figure 6B), and was most prolonged with combined TSA+FBK exposure (Figure 6D and Figure S13). Chromatin immunoprecipitation assays showed an increase in acetylated histone H3 DNA binding at the *hPRL* promoter following all treatments, with the highest level induced by TSA (Figure 7).

**Figure 8:**
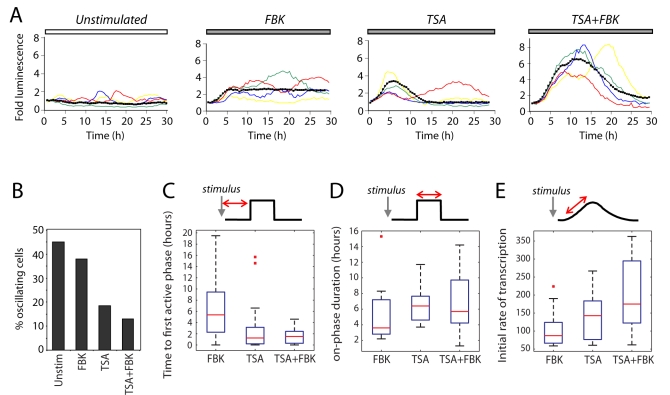
Kinetics of prolactin transcription bursts. (A) The colored lines show luminescence data from the *hPRL-Luc* reporter gene in single representative GH3-DP1 cells in unstimulated, FBK, TSA, or TSA+FBK conditions. The thick black line in each graph shows the average from one experiment (unstimulated, *n* = 15 cells; FBK, *n* = 40 cells; TSA, *n* = 21 cells; TSA+FBK, *n* = 27 cells). (B) The effect of the treatments in (A) on the persistence of oscillations within a 30 h period. The binary model was used to quantify (C) the time to first on-phase, (D) duration of active on-phase, and (E) initial rate of transcription following the first activation after FBK, TSA, or TSA+FBK treatments.

Analysis of gene expression kinetics from the *hPRL-Luc* reporter gene in GH3-DP1 cells showed that the transcriptional cycles persisted following FBK treatment. However, they were only seen in less than 20% of cells treated with TSA (Figure 8A and B). Analysis with the binary switch model showed that when the cells were treated with FBK, the median time to activation was longer and more variable than with TSA or TSA+FBK (Figure 8C). This supports the hypothesis of a refractory period of transcription inactivation in which chromatin remodeling may play an important role. Treatment with TSA increased the duration of the on-phase and the initial rate of transcription (Figure 8D and E). Combined TSA+FBK treatment increased the transcription rate during the on-phase following activation (Figure 8E), resulting in a pronounced increase in maximum reporter gene expression (Figure 8A). The response of cells to treatments that included TSA was more rapid and coordinated, suggesting that histone acetylation has a key role in the coordination of the temporal kinetics of transcription. Transcription of the *hPRL* gene might therefore require a long period of chromatin remodeling that is the source of the observed refractory phase.

### Cycles of Prolactin Transcription Are Enhanced by a Non-Random Refractory Period in the Off-Phase

**Figure 9:**
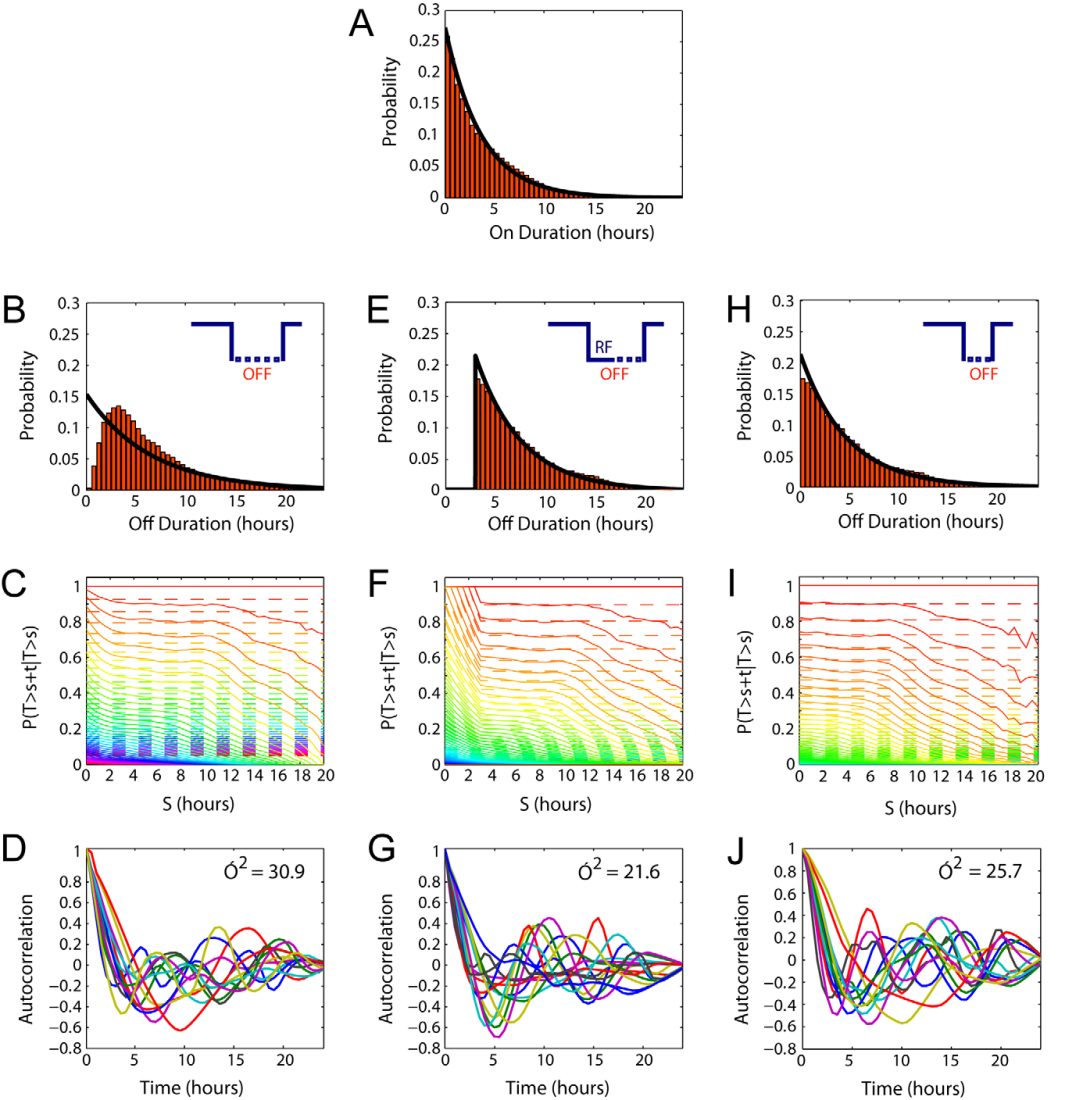
Non-random timing of off-phases increases the cyclicity of transcriptional cycles. Histograms showing the distribution of (A) on-times, (B) off-times, (E) off-times greater than 3 h with the refractory period, and (H) off-times greater than 3 h without the refractory period, estimated from the Markov Chain Monte Carlo algorithm (Text S1 Section 3.4). The superimposed black lines show the fit of an exponential distribution with the same mean value as the data. (C, F, and I) The off-phases in the system are not memoryless. The probability of having to wait for t hours in the off state given that the off-time has already lasted for s hours is plotted for a range of values of t for the distributions in (B, E, and H), respectively. The dashed lines represent the exponential probabilities, and the solid lines are the sample probability estimates. The uppermost lines are calculated when t = 0, the lines beneath that are calculated for t = 0.5, and so on in increments of 0.5. In a memoryless system such as that described by the telegraph process this is independent of s (hence s is constant for a given value of t), but for our system this probability decreases significantly with s during the refractory period (F). The decrease of this probability for higher values of s is due to the finite length of our time-series. (D, G, and J) Autocorrelation functions for a number of mRNA time-series simulated using on and off durations selected at random from the distributions above (B, E, and H, respectively). The variance of the time of the first peak (which estimates period) is given in each plot. In (E) the refractory period is indicated by RF.

The rate at which mRNA is transcribed can be affected by different molecular mechanisms, including binding and dissociation of transcription factors, spatial reorganization, and/or chromatin remodeling. Previous studies [5],[10],[26] have considered a model in which the fluctuations of transcription rates are caused by overall dynamics that can be described by a “random telegraph process,” where the gene switches between an active and an inactive state:
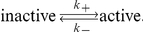
The mean residence times for the active and inactive states are *t_active_* = 1/*k*_−_ and *t_inactive_* = 1/*k*_+_ and switch on- and off-times are drawn from an exponential distribution with means *t_active_* and *t_inactive_*. For the on-times our results fit this hypothesis and the estimated distribution of on-times is exponential (Figure 9A). Such a system would be memoryless in that the time already spent waiting in that state would not affect how much longer one would have to wait until the switch (Text S1 Section 3.5). The distribution of off-times in our data strongly contradicted this and was not distributed exponentially. This is shown in Figure 9B in which the exponential distribution (black line) is a poor fit of the data. We found that the system had a definite memory, where the length of time already spent in the inactive state affected the length of time remaining in that state (Figure 9C). Thus the dynamics of these transcription cycles are not compatible with the mathematical models previously derived from analysis of single cell RNA counting [10].

An MCMC algorithm was applied which gave a distribution of estimates for the off-phase durations for single cells. When these estimates were amalgamated into a population distribution, the most likely off duration was at 3 h (Figure 9B), which is consistent with the individual estimates in Figure 5. Thus, the most likely explanation of the memory effect was the existence of a refractory period of approximately 3 h (Figure 5C and D). If this refractory period is enforced, by removing any chance of an off duration of less than 3 h, then the excess off durations were distributed approximately exponentially (Figure 9E). This refractory period means that the system still has a memory (Figure 9F). Removing the refractory period (Figure 9H) meant that the system became memoryless (Figure 9I).

One effect of this memory or refractory period is to cause more cyclicity than would be seen in a telegraph process. In a system with a 3 h refractory period where the excess off-time is exponentially distributed (Figure 9E), a higher proportion of the off-times would be just over 3 h and the system would appear more cyclic. To quantify the regularity of the transcription cycles, we simulated 1,000 cells with on and off durations drawn from the corresponding distributions. We then performed autocorrelation analysis on this simulated data set and the variance in the timing of the first peak was taken to be our measure of cyclicity. The variance in first peak timing was higher when there was no refractory period (Figure 9D and J) than when a refractory period was enforced (Figure 9G). This analysis revealed that the presence of a defined refractory phase increases the regularity of the transcription cycles.

## Discussion

Physiologically important hormones such as PRL may be subject to both acute short-term regulation and long-term seasonal control. This could be achieved at the individual cell level by graded gene expression with feedback control of PRL expression. Such a model would suggest that each cell would express similar levels of PRL. Alternatively individual cells could dynamically switch between on- and off-phases producing a stable population average level of prolactin expression across the whole tissue. Studies of PRL promoter activity in intact pituitary tissue showed the whole tissue response was synchronized, although adjacent cells were not coordinated [27].

We have previously shown that expression from the PRL promoter is heterogeneous over time in individual cells from cell lines [14] and more recently in intact pituitary tissue [27]. The latter study suggested that isolated cells show greater heterogeneity than cells in tissue but that tissue-level cellular heterogeneity is still important. In a different study we recently described data that suggested that cellular heterogeneity may be genetically encoded by the timing of negative feedback loops in the NF-κB signaling system and that this may lead to out-of-phase oscillations in NF-κB signaling between cells [28]. This study raised the hypothesis that cellular heterogeneity may in fact be advantageous, leading to more robust tissue-level responses. (Studies in other systems are in support of the idea that cellular variability is advantageous; e.g. [29].) The present study quantifies the level of heterogeneity in the dynamics of PRL gene expression in single cells. This heterogeneity may ensure stability in gene expression at the tissue level, while ensuring the readiness of the tissue as a whole to respond rapidly to signals.

These data suggest that the overall level of *hPRL* transcription in pituitary tissue may be determined by three variables: (1) the frequency of transcriptional bursts, (2) the duration of the on-phase, and (3) the rate of transcription during the on-phase. Our studies suggest that within a population of cells there is a continuous transition from an activated “on” state to a basal “off” or “low” state, with an overall cycle of around 11 h. This cycle is longer than previously described transcriptional cycles/pulses [10]–[12], with a different structure due to the presence of a defined refractory period of transcriptional inactivation. Because of the much longer time-scales involved, the source of the stochasticity would not be expected to derive directly from that due to random molecular processes involving small molecule numbers. However, within this cycle, the average transcriptional on-phase is ∼4 h, which is closer to previously defined transcriptional bursts and cycles [24]. The majority of the overall cycle time described here is therefore dominated by the off-phase. This could be due to a repressive chromatin state or alternatively could be regulated by chromosome topology with the timing dependent on the movement of the genes into and out of transcription factories [30]–[32].

**Figure 10:**
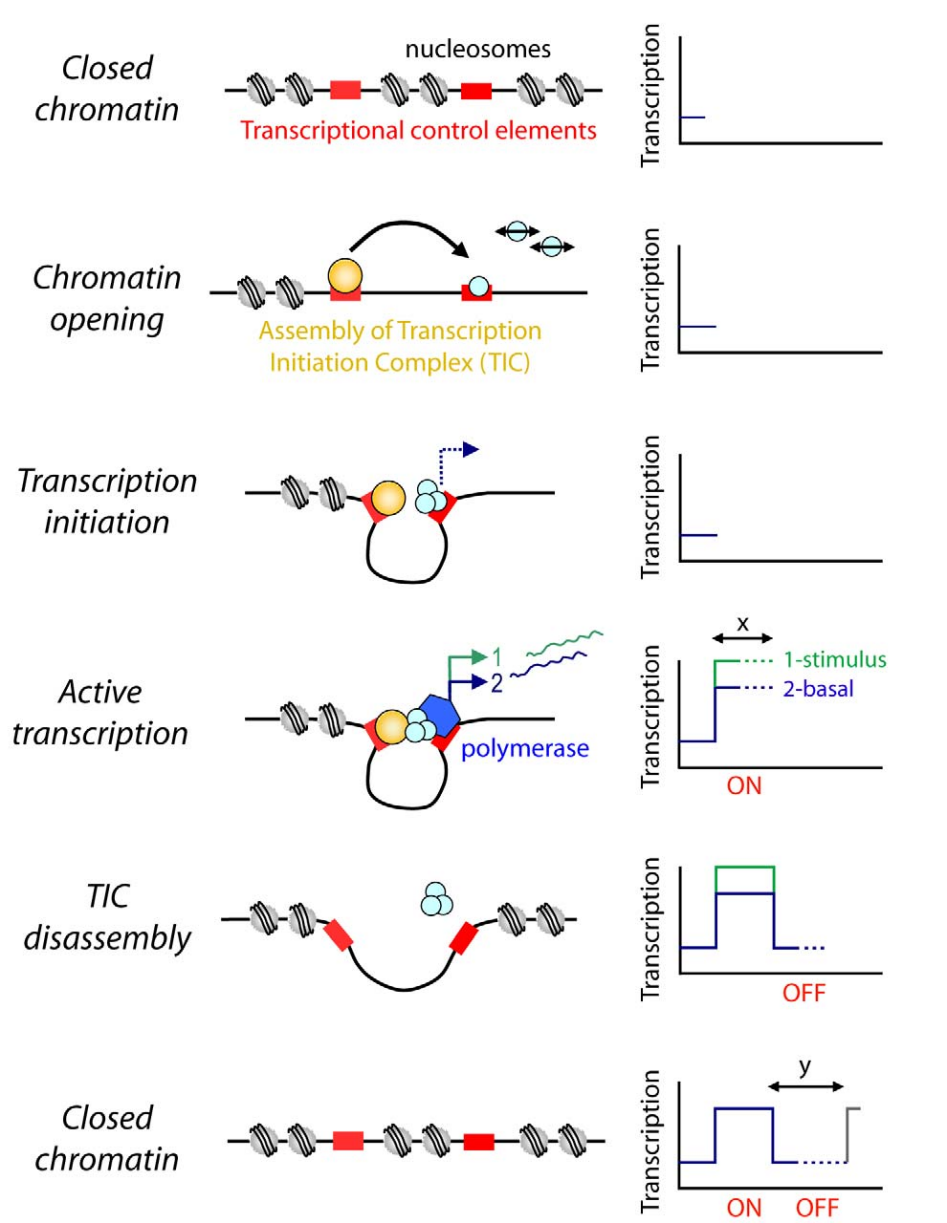
Generation of transcription cycles. The schematic diagram proposes a mechanistic model whereby chromatin remodeling processes generate the binary on and off stochastic cycles of transcription. χ and y denote phases of variable duration.

Although the transcription cycle maintains relatively defined dynamics, the timing of transcription cycles from two independent promoters within a cell were heterogeneous, indicative of a system where intrinsic noise generated by local chromatin dynamics dominates extrinsic noise. Strong correlation between promoters could only be achieved following disruption of chromatin, suggesting that the cycles of *hPRL* transcription might involve epigenetic cycles of histone acetylation and deacetylation (Figure 10). Thus, an independent chromatin-regulated cycle of gene activity may occur at each locus. Cycles in the binding of transcription factors and polymerase at certain genes in the nucleus have been observed following oestrogen stimulation [24],[33],[34]. In that system, a refractory period also seems to occur [24],[34], as well as cycles of epigenetic chromatin modification [33].

The current study illustrates the importance of new integrated experimental and mathematical approaches for dynamic single cell analyses. Providing high-frequency time-lapse imaging data of the same cells over long time periods enabled the identification of dynamic transcription processes, phenomena which are invisible to the single cell RNA counting snapshots used for previous analyses of transcriptional bursting [35],[36]. Biologically meaningful model parameters can be directly measured from the imaging data without requiring prior assumptions about the nature of the timings inherent in the system. As such, models fitted to these data accurately represent the underlying processes that lead to the time-course data.

The data described in this study and by others [37] suggest that transcription cycles might emerge de novo from the intrinsic kinetics of the processes of transcription initiation, elongation, and termination. In our study we find that a transcriptional refractory period can have a dominant effect that leads to increased regularity in the timing of transcriptional cycles. This is in marked contrast with other cellular oscillatory systems such as NF-κB [38],[39], p53 [40], Erk2 [41], and the circadian clock [42] where negative feedback loops are believed to lead to the oscillatory dynamics with varying frequencies [43]. In particular in the NF-κB system, where a transcriptional delay (refractory period) of 45 min in IkBε activation following TNFα stimulation leads to cell-to-cell heterogeneity through the precise timing of this feedback loop [38]. This transcriptional delay is precisely timed to maximize the effect of IkBε transcriptional noise on ensuring out-of-phase oscillations [28].

A key question is whether the signals that activate *hPRL* in vivo are themselves graded or pulsatile. *hPRL* is itself under the control of NF-κB [44], which responds dynamically to pulsatile cytokine stimulation [38]. Recently, glucocorticoid receptor, which also regulates *hPRL* expression [44], has also been shown to cycle in response to pulsatile stimulation [45]. Although the secreted hormone PRL is stored in secretory granules and displays both pulsatile and circadian patterns, is it not yet clear how transcription relates to secretory events in individual cells.

This system may be a new paradigm for understanding gene expression dynamics in vivo and may be important for understanding natural cell-to-cell variation in protein levels [46],[47]. We have previously shown that gene promoter activity in lactotroph cells within pituitary tissue is non-uniform, with varying expression levels from adjacent cells [27]. However, these stochastic patterns together provide tissue-wide long-term coordinated behavior. If we include the new information gained in this present article we can start to build up a picture of a mosaic tissue structure, where, at any one time, a subset of cells are expressing *PRL*, a subset are in an inactive and refractory state, and a further subset are in an activatable state, ready to respond to a stimulus. A transient hormonal stimulus to the tissue would recruit this last subset of cells immediately, whereas sustained stimulus would progressively recruit additional cells exiting the refractory phase, resulting in a more sustained increase in *PRL* expression. If these stochastic and cyclical patterns of gene expression occur normally in intact tissue, such a mechanism would facilitate highly flexible transcriptional responses, allowing tissues to mount either acute or chronic responses to environmental cues, while maintaining a controlled average level of gene expression in the resting state.

## Materials and Methods

### Materials

All animals were handled in strict accordance with U.K. Home Office License regulations and subject to local ethical committee review.

Fetal calf serum (FCS) was from Harlan Sera-Lab, Crawley Down, U.K. Luciferin was from Bio-Synth, Switzerland. Forskolin, BayK-8644, and Trichostatin A were all from Sigma, U.K.

### Production of Stable Cell Lines and Cell Culture

The GH3-DP stable cell lines were generated by incorporating a 5 kb *hPRL-d2EGFP* reporter gene into the previously described GH3/*hPRL-Luc* cell line [14]. This was co-transfected with a hygromycin-selectable plasmid to enable antibiotic clonal selection. Generation of stable BAC-transfected rat pituitary GH3 cells containing the 160 kb *hPRL-Luc* gene was described previously [16]. GH3-DP1 cells, GH3-BAC cells, and collagenase type I dispersed primary pituitary cells were cultured in DMEM containing 10% FCS and maintained at 37°C 5% CO_2_.

### Generation of Dual Reporter Transgenic Primary Rat Pituitary Cells

Generation of BAC-transgenic rats expressing luciferase and d2EGFP under the control of identical *hPRL* 160 kb genomic fragments was described previously [16]. Transgenic line 37 was a luciferase-BAC transgenic rat line which was found to have two single reporter insertion sites. Further breeding of this line was undertaken to create two separate transgenic lines each with single integration sites. Line 37A had a single autosomal integration site, and line 37B an X-chromosomal integration site. (These lines were the source of the pituitary cells used in Figure 1.) Independently, a destabilized EGFP reporter transgenic rat line was constructed, termed line 455, which also had a single insertion site. The copy number of the luciferase-BAC transgene in line 37A was measured as ≤2 and that of the d2EGFP line 455 was ≤5. In order to generate the dual reporter rat cell line (previously referred to as PRL-Luc/d2eGFP in [16]), line 37A was crossed with line 455.

### Luminescence Imaging

GH3-DP1 cells were seeded in 35 mm glass coverslip-based dishes (IWAKI, Japan) 20 h prior to imaging. Luciferin (1 mM) was added at least 10 h before the start of the experiment, and the cells were transferred to the stage of a Zeiss Axiovert 200 equipped with an XL incubator (maintained at 37°C, 5% CO2, in humid conditions, carefully monitored to ensure equivalent conditions to a standard cell incubator) maintained within a dark room. Luminescence images were obtained using a Fluar ×20, 0.75 NA (Zeiss) air objective and captured using a photon-counting charge coupled device camera (Orca II ER, Hamamatsu Photonics, U.K.). Bright field images were taken before and after luminescence imaging to allow localization of cells. Sequential images, integrated over 30 min, were taken using 4 by 4 binning and acquired using Kinetic Imaging software AQM6 (Andor, Belfast, U.K.). In the relevant experiments, 5 µM forskolin and 0.5 µM BayK-8644 (FBK), 30 ng/ml TSA, or both stimuli were added directly to the dish at the indicated times.

### Fluorescence Imaging

Cells were prepared and imaged using the conditions and microscope described above. Excitation of d2EGFP was performed using an argon ion laser at 488 nm. Emitted light was captured through a 505–550 nm bandpass filter from a 545 nm dichroic mirror. Data were captured and analyzed using LSM510 software with consecutive autofocus.

### Alternate Longitudinal Imaging of Fluorescence and Luminescence

Cells were prepared and visualized using confocal microscopy as described above. A single field of cells was selected and five sequential fluorescence images were captured using autofocus. After a 10 min delay, the microscope and surrounding light-emitting devices were turned off or covered and a single luminescence image was captured using a cooled CCD camera (30 min integration). The equipment was then restarted (taking 5 min), and after a 10 min delay (to ensure laser stability), fluorescence images were taken. This hourly cycle was repeated for up to 21 h.

### ChIP Assays and RT-PCR

GH3/hPRL-luc (D44) cells (3×10^6^) were plated in 10 cm^2^ dishes and left for 40 h. Dishes were treated for 2 h (unstimulated, FBK, TSA, TSA+FBK) and then ChIP assays were performed as described previously [38] based on the protocol by Upstate Biotechnology.

Immunoprecipitation was carried out using 5 µg of either Anti-Acetylated H3 or Anti-IgG antibodies (Upstate Biotechnology). DNA was extracted and amplified by PCR as described previously [38]. The following primer sequences were used: hPRL Promoter1 left GCAATCTTGAGGAAGAAACTTGA, right AGGCATTCGTTTCCCTTTTC amplifying 347 bp of DNA. PCR products were resolved using agarose gel electrophoresis and were analyzed by AQM Advance 6.0 software (Kinetic Imaging, U.K.).

### Analysis of Imaging Data

Analysis was carried out using Kinetic Imaging AQM6 software (Andor). Regions of interest were drawn around each single cell, and mean intensity data were collected. Data were collected from every single cell within the field. The average instrument dark count (corrected for the number of pixels being used) was subtracted from the luminescence signal. In dual reporter experiments, cells dividing within the experiment were eliminated from the analysis. For single reporter experiments, analysis ceased at the point of cell division. For the GH3 cell line, the cell cycle time is approximately 40 h [14],[18].

### Inference of Transcription Models

We use the following ordinary differential equations model for the reconstruction of transcription profiles from protein data (see also [23], Text S1 Section 3.2).
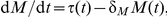


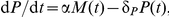
where *M* and *P* denote concentration of reporter mRNA and protein, respectively. The first equation describes the dynamics of mRNA molecules with transcription function τ*(t)* and degradation rate δ*_M_*. Protein is synthesized at a rate proportional to the abundance of mRNA and is degraded at rate δ*_P_*. The various parameters will be different for the d2EGFP and Luc reporters. The transcription profile can be reconstructed via
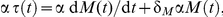
where the unobserved mRNA profile is expressed as a function of the observed solution path of *P(t*), i.e. α*M(t)* = d*P*/d*t*+δ*_P_P(t)*. Since *M* is not observed, prior knowledge of the rates δ*_M_* and δ*_P_* is necessary for the identification of the transcription profiles. We estimated these from two separate experiments where translation of reporter protein was inhibited by adding cycloheximide and transcription was inhibited by adding Actinomycin D (see Text S1 Sections 1 and 3.2.2). The rates estimated for δ*_M_* and δ*_P_* associated with d2EGFP and Luc are treated as known parameters. For inference on the transcription profile, the solution path *P(t)* is approximated by a flexible continuous function, here a spline representation, fitted to the observed protein data (Text S1 Section 3.2.3). The transcription profile is then reconstructed using the discrete Euler approximation to the differential equations for a small time interval, replacing *P(t)* by the fitted continuous function. In order to study the correlation in transcription of the dual reporter constructs within a cell, we compute the rank correlation coefficient between the two reconstructed transcription profiles of the two reporters d2EGFP and Luc within a cell (Text S1 Section 3.3). As this may vary over time (in particular for stimulated experiments), all correlations are computed as a function of the length of time since a stimulus (TSA, FBK) was added starting from 1.5 h (to allow for a reasonable minimal length over which any correlation is computed) to 8 h. For unstimulated experiments we computed correlations after 2 h into the experiment to avoid any initial bias. The question of estimating and including protein maturation times is addressed in [48]. In calculating the correlations, the relevant quantity is the difference in maturation times between the two reporters. We have therefore included this process with a constant difference of up to 1 h. We have verified that such delays do not change our correlation results. It is clear from this that if we instead assumed an exponentially distributed delay with a similar difference in means, then this would not affect the correlation results.

## Supporting Information

Figure S1 Prolactin promoter activity is pulsatile. Prolactin promoter activity was assessed in pituitary GH3 cells stably transfected with 5 kbp *PRL* promoter-luciferase reporter protein (GH3-DP1 cells; A, B) and GH3 cells containing a 160 kbp *PRL* BAC-luc construct (C). Each line represents a single cell where the first peak of each cell is aligned to time zero. Peak frequency (cycle length) and signal intensity are compared between the two cell lines and primary cell cultures from *PRL*-BAC-luc transgenic rats (D). Bars show standard deviation from at least 42 cells in three experiments per cell type. Colored regions on schematic promoter-reporter constructs represent 5′- or 3′-flanking regions (grey), luciferase reporter sequence (red), and h*PRL* exons 1a and 2–5 (yellow, not to scale).
(0.24 MB PDF)

Figure S2 Transgene copy number in a dual reporter stable cell line. (A) Stable GH3 cell line expressing luciferase and d2EGFP reporters both under the control of the 5 kbp human prolactin promoter (GH3-DP). The copy number of each reporter was quantified using absolute quantification real-time PCR. Standard curves of known plasmid concentrations were generated for 5 kb *PRL*-luc (B) and 5 kb *PRL*-d2EGFP (C). Sequences of luciferase and d2EGFP were amplified from genomic DNA extracted from a known quantity of GH3-DP cells and copy number was determined by comparison to plasmid standards (dotted lines on graphs represent ct value from 5,000 cells).
(0.13 MB PDF)

Figure S3 Detection of single cell gene expression in GH3-DP1 cells, using the *PRL*-d2EGFP reporter construct. Possible hypothetical models of binary or graded transcriptional response are shown in green (upper panels), and experimental data are shown in the bottom panel. Flow cytometry indicated that the combined stimulus of FBK (5 µM forskolin and 0.5 µM BayK-8644) significantly increased the expression of *PRL*-d2EGFP. A biphasic population was detected under control conditions. Stimulation induced a significant increase in EGFP transcription, with increasing proportions of cells displaying high signal, and the bi-modality of the population persisted over at least 12 h.
(0.09 MB PDF)

Figure S4 Two fields of cells from separate experiments showing transmitted light images (left panels), fluorescence images (middle panels), and luminescence images (right panels) from GH3-DP1 cells. Regions of interest show single cells used for analysis. No correlation was detected between the signal intensity of the two reporters in single cells, as indicated by scatter plots where each dot represents a single cell.
(0.66 MB PDF)

Figure S5 Time line outlining the process of capturing sequential fluorescence and luminescence images from the same single cells. Numbers represent time in minutes.
(0.07 MB PDF)

Figure S6 Example plots from 17 single cells showing fluorescence (green) and luminescence (red) h*PRL* promoter-driven reporter construct data in unstimulated conditions over 21 h. Bottom right-hand graph shows the average fluorescence and luminescence traces from a field of cells.
(0.20 MB PDF)

Figure S7 The effect of various stimuli and combinations of stimuli on expression of *PRL* were assessed using luminometry. (A, B) 10 ng/ml TNFa, 5 µM forskolin (FSK), and 0.5 µM BayK-8644 (BayK) were used. (C) The effect of TSA (30 ng/ml) and TSA in combination with 5 µM FSK and 0.5 µM BayK (FBK).
(0.16 MB PDF)

Figure S8 Sample ACFs of protein time series from Luc reporter constructs. Time series as in Figure 1 (B,C,E,F) of the main text. The *x*-axis gives the delay $s$ in hours. Each single sample ACF corresponds to the time profile observed for a single cell. Top left: Sample ACFs of time series displayed in Figure 1B of the main text (DP1, results for 15 cells, 25 hourly observations). Top right: Sample ACFs of time series displayed in Figure 1C of the main text (BAC, results for 17 cells, 95 half-hourly observations). Bottom left: Sample ACFs of time series displayed in Figure 1E of the main text (37 B, results for 24 cells, 185 half-hourly observations). Bottom right: Sample ACFs of time series displayed in Figure 1F of the main text (37 A, results for 20 cells, 95 half-hourly observations). The two bottom experiments have many cells showing longer oscillations around 25–35 h. In the experiment displayed in the bottom left graph one can see that 3 out of the 24 cells behave like a white noise process.
(2.38 MB PDF)

Figure S9 Sample ACFs of reconstructed transcription profiles from Luc (left) and d2EGFP (right) reporter constructs. The reconstruction profiles were computed using a spline approach (described below) on a fine grid of 0.1 h using the estimated posterior mean of the spline coefficients. (a) Dual experiment C1-unstim2 (21 cells, 14 h). (b) Dual experiment C1-unstim1 (29 cells, 15 h). (c) Dual experiment C2-unstim1 (21 cells, 15 h). (d) Dual experiment C2-unstim2 (15 cells, 14 h). (e) Dual experiment C1-unstim4 (20 cells, 21 h). See Table S2 for a list of dual experiments.
(4.19 MB PDF)

Figure S10 This figure shows the fit of the differential equations to the degradation data for Luc (left) and d2EGFP (right). The bottom panel shows the fit of Equation 5 to average protein data from experiments (A). The top panel shows average reconstructed mRNA profiles from protein data experiments (B) and the fit of equation (5) to the reconstructed mRNA profiles.
(0.21 MB PDF)

Figure S11 Reconstruction of transcription profile from protein data (green, d2EGFP; red, Luc) for four randomly selected cells. The *y*-axis is in arbitrary units. The results for each cell are shown in a panel of three plots. Left, reconstructed transcription profile; middle, reconstructed mRNA profile; right, observed protein data (dots) together with the spline fit to the protein data.
(0.30 MB PDF)

Figure S12 Correlation plot for pooled groups: DP1 (83 cells, 3 top left panels), DP2 (36 cells, 3 top right panels), and primary (22 cells, 3 bottom panels). Each set of three panels as follows. Top panel, correlation (a) between reconstructed transcription of Luc and d2EGFP reporter; middle panel, correlation of reconstructed transcription of Luc reporter between cells within the same experiment (b); bottom panel, correlation of reconstructed transcription of d2EGFP reporter between cells within the same experiment (c). *x*-axis, time length over which correlation is computed; *y*-axis, (rank) correlation coefficient. For given time length each boxplot summarizes the distribution of the estimated correlation over the population of cells in the group by the estimated 0.025, 0.25, 0.5, 0.75, and 0.975 quantiles. The solid line gives the estimated median of each boxplot, and the dashed lines give the 95% interval for the median (points are only connected between boxplots for purpose of illustrating the trend). All top panels giving the correlation between reporter constructs are presented in the main text (confidence intervals differ slightly as they are estimated from another set of B = 4,000 bootstrap samples).
(0.09 MB PDF)

Figure S13 Correlation plot for pooled groups: unstim (119 cells, three top left panels), FBK (87 cells, three top right panels), TSA (74 cells, three bottom left panels), and TSA+FBK (41 cells, three bottom right panels). All other explanations as in Figure S12.
(0.11 MB PDF)

Figure S14 Correlation analysis for time-shifted GFP to allow for the difference d in maturation time between GFP and Luciferase. Left, d = 0.5 h. Right, d = 1 h. Row 1, TSA+FBK. Row 2, TSA. Row 3, FBK. Row 4, Unstimulated. All other explanations as in Figure S12.
(0.05 MB PDF)

Figure S15 The distribution of off-times without weak switches removed.
(0.03 MB PDF)

Figure S16 Estimated distributions of the switch times (in hours). In this case, the cell in question was determined to have three switches, and each color corresponds to the estimates for an individual switch time.
(0.04 MB PDF)

Figure S17The estimated distributions of 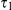, 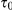, and 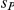.(0.84 MB PDF)

Table S1Results of degradation rate estimation. Estimated 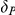 and 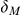
(posterior standard errors in brackets) for Luc (left) and d2EGFP (right). All rates are per hour. If data were used for more than one experiment, the average estimate is used (stated in bold) for the subsequent reconstruction of transcription and for fitting the switch model.(0.06 MB PDF)

Table S2 List of dual reporter experiments. For the correlation analysis, data from unstimulated experiments were pooled according to cell type into DP1, DP2, and primary. Data were also pooled according to stimulus into unstim, FBK, TSA, and TSA+FBK. Column 4 gives time of stimulation for stimulated experiments. Column 5 gives number of cells per experiment used for analysis after discarding cells with very low amplitude (number before discarding in brackets). The final column gives number of data points measured at hourly intervals. Data for the two reporters are not taken at identical time points, but this is corrected for in the analysis.
(0.03 MB PDF)

Text S1 Description of experimental and theoretical methodology.
(0.25 MB PDF)

## Abbreviations

BAC: bacterial artificial chromosome
d2EGFP: destabilized enhanced green fluorescent protein
FBK: forskolin and BayK 8644
FSK: forskolin
hPRL: Human prolactin
hPRL-Luc: Human prolactin–Luciferase
hPRL-d2EGFP: human prolactin-Destabilized Enhanced Green Fluorescent Protein
MCMC: Markov Chain Monte Carlo
TSA: trichostatin A
