# Supplementary material for: Dynamic Analysis of Stochastic Transcription Cycles
Source: PLoS Biol. 2011 Apr 12;9(4):e1000607. doi: 10.1371/journal.pbio.1000607 (PMC3075210; doi:10.1371/journal.pbio.1000607)
Supplement: Figure S1 — Prolactin promoter activity is pulsatile. Prolactin promoter activity was assessed in pituitary GH3 cells stably transfected with 5 kbp PRL promoter-luciferase reporter protein (GH3-DP1 cells; A, B) and GH3 cells containing a 160 kbp PRL BAC-luc construct (C). Each line represents a single cell where the first peak of each cell is aligned to time zero. Peak frequency (cycle length) and signal intensity are compared between the two cell lines and primary cell cultures from PRL-BAC-luc transgenic rats (D). Bars show standard deviation from at least 42 cells in three experiments per cell type. Colored regions on schematic promoter-reporter constructs represent 5′- or 3′-flanking regions (grey), luciferase reporter sequence (red), and hPRL exons 1a and 2–5 (yellow, not to scale). (0.24 MB PDF) [file pbio.1000607.s001.pdf]

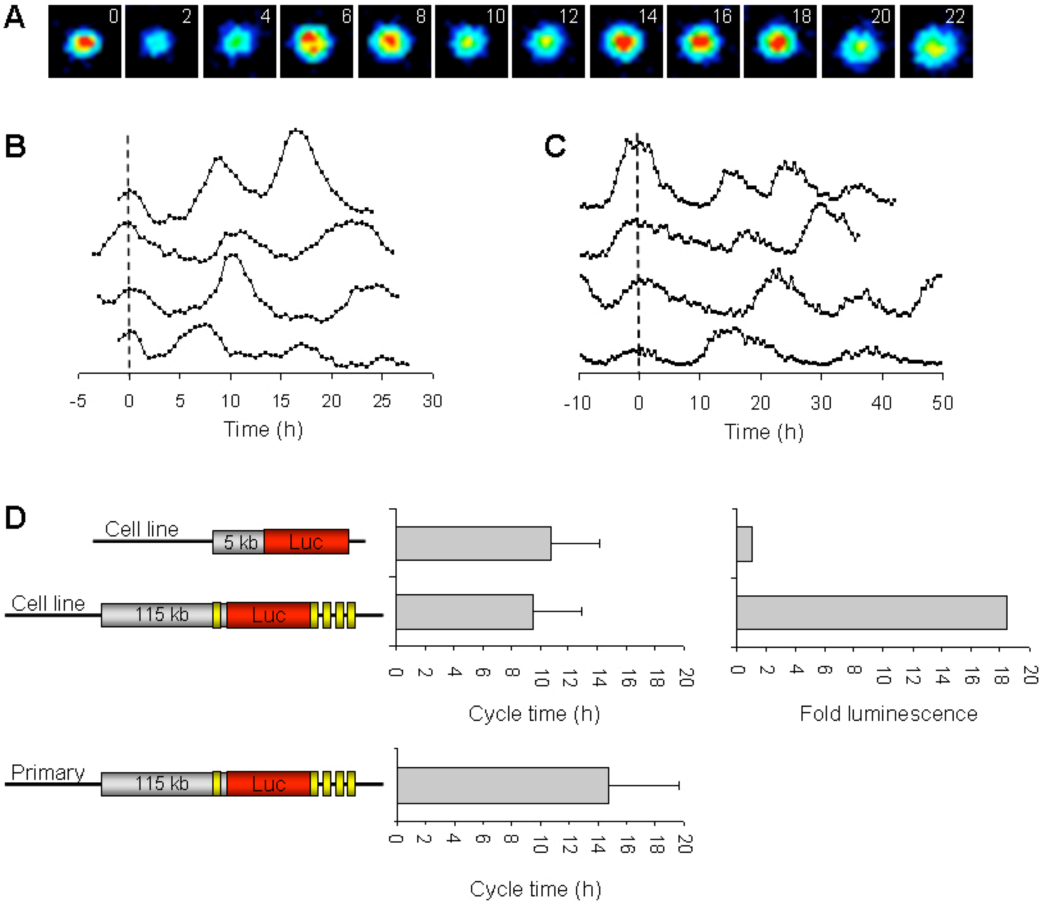

Fig. S1: Prolactin promoter activity is pulsatile. Prolactin promoter activity was assessed in pituitary GH3 cells stably transfected with 5kbp *PRL* promoter-luciferase reporter protein (GH3-DP1 cells; A, B) and GH3 cells containing a 160kbp *PRL* BAC-luc construct (C). Each line represents a single cell where the first peak of each cell is aligned to time zero. Peak frequency (cycle length) and signal intensity are compared between the two cell lines and primary cell cultures from *PRL*-BAC-luc transgenic rats (D). Bars show standard deviation from at least 42 cells in 3 experiments per cell type. Colored regions on schematic promoter-reporter constructs represent 5′- or 3′-flanking regions (grey), luciferase reporter sequence (red), and h*PRL* exons 1a and 2-5 (yellow, not to scale).
