# Supplementary material for: Dynamic Analysis of Stochastic Transcription Cycles
Source: PLoS Biol. 2011 Apr 12;9(4):e1000607. doi: 10.1371/journal.pbio.1000607 (PMC3075210; doi:10.1371/journal.pbio.1000607)
Supplement: Figure S2 — Transgene copy number in a dual reporter stable cell line. (A) Stable GH3 cell line expressing luciferase and d2EGFP reporters both under the control of the 5 kbp human prolactin promoter (GH3-DP). The copy number of each reporter was quantified using absolute quantification real-time PCR. Standard curves of known plasmid concentrations were generated for 5 kb PRL-luc (B) and 5 kb PRL-d2EGFP (C). Sequences of luciferase and d2EGFP were amplified from genomic DNA extracted from a known quantity of GH3-DP cells and copy number was determined by comparison to plasmid standards (dotted lines on graphs represent ct value from 5,000 cells). (0.13 MB PDF) [file pbio.1000607.s002.pdf]

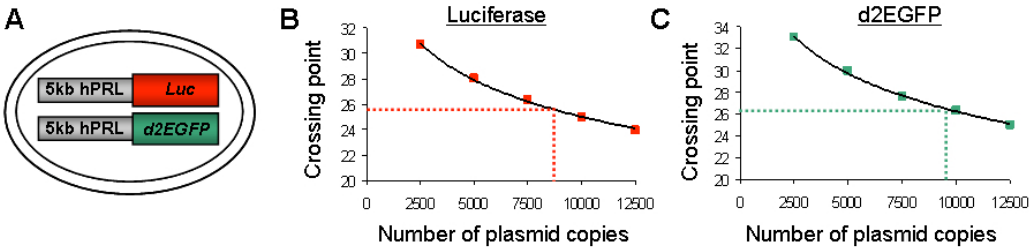

Fig. S2: Transgene copy number in a dual reporter stable cell line. (A) Stable GH3 cell line expressing luciferase and d2EGFP reporters both under the control of the 5 kbp human prolactin promoter (GH3-DP). The copy number of each reporter was quantified using absolute quantification real-time PCR. Standard curves of known plasmid concentrations were generated for 5kb *PRL*-luc (B) and 5kb *PRL*-d2EGFP (C). Sequences of luciferase and d2EGFP were amplified from genomic DNA extracted from a known quantity of GH3-DP cells and copy number was determined by comparison to plasmid standards (dotted lines on graphs represent ct value from 5000 cells).
