# Supplementary material for: Dynamic Analysis of Stochastic Transcription Cycles
Source: PLoS Biol. 2011 Apr 12;9(4):e1000607. doi: 10.1371/journal.pbio.1000607 (PMC3075210; doi:10.1371/journal.pbio.1000607)
Supplement: Figure S3 — Detection of single cell gene expression in GH3-DP1 cells, using the PRL-d2EGFP reporter construct. Possible hypothetical models of binary or graded transcriptional response are shown in green (upper panels), and experimental data are shown in the bottom panel. Flow cytometry indicated that the combined stimulus of FBK (5 µM forskolin and 0.5 µM BayK-8644) significantly increased the expression of PRL-d2EGFP. A biphasic population was detected under control conditions. Stimulation induced a significant increase in EGFP transcription, with increasing proportions of cells displaying high signal, and the bi-modality of the population persisted over at least 12 h. (0.09 MB PDF) [file pbio.1000607.s003.pdf]

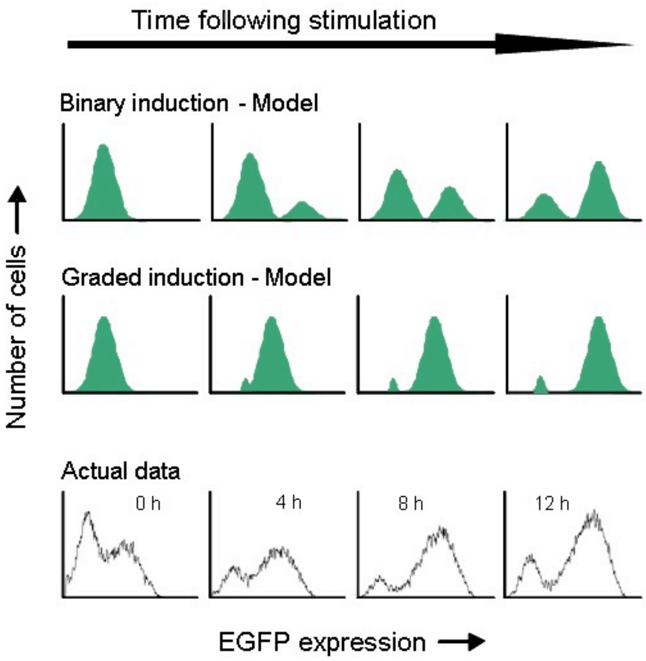

Fig. S3: Detection of single cell gene expression in GH3-DP1 cells, using the *PRL*-d2EGFP reporter construct. Possible hypothetical models of binary or graded transcriptional response are shown in green (upper panels), and experimental data are shown in the bottom panel. Flow cytometry indicated that the combined stimulus of FBK (5μM forskolin and 0.5μM BayK-8644) significantly increased the expression of *PRL*-d2EGFP. A biphasic population was detected under control conditions. Stimulation induced a significant increase in EGFP transcription, with increasing proportions of cells displaying high signal, and the bi-modality of the population persisted over at least 12h.
