# Supplementary material for: Dynamic Analysis of Stochastic Transcription Cycles
Source: PLoS Biol. 2011 Apr 12;9(4):e1000607. doi: 10.1371/journal.pbio.1000607 (PMC3075210; doi:10.1371/journal.pbio.1000607)
Supplement: Figure S4 — Two fields of cells from separate experiments showing transmitted light images (left panels), fluorescence images (middle panels), and luminescence images (right panels) from GH3-DP1 cells. Regions of interest show single cells used for analysis. No correlation was detected between the signal intensity of the two reporters in single cells, as indicated by scatter plots where each dot represents a single cell. (0.66 MB PDF) [file pbio.1000607.s004.pdf]

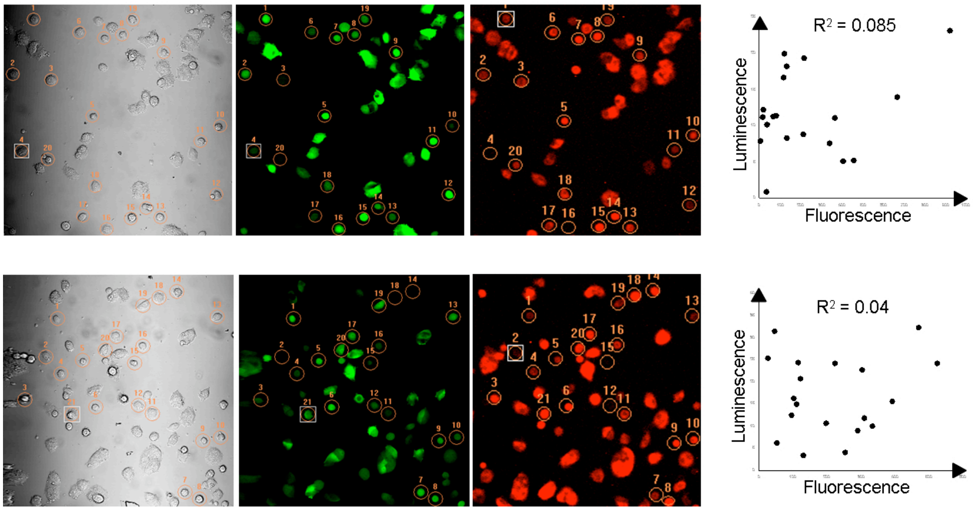

Fig. S4: Two fields of cells from separate experiments showing transmitted light images (left panels), fluorescence images (middle panels) and luminescence images (right panels), from GH3-DP1 cells. Regions of interest show single cells used for analysis. No correlation was detected between the signal intensity of the two reporters in single cells, as indicated by scatter plots where each dot represents a single cell.
