# Supplementary material for: Dynamic Analysis of Stochastic Transcription Cycles
Source: PLoS Biol. 2011 Apr 12;9(4):e1000607. doi: 10.1371/journal.pbio.1000607 (PMC3075210; doi:10.1371/journal.pbio.1000607)
Supplement: Figure S5 — Time line outlining the process of capturing sequential fluorescence and luminescence images from the same single cells. Numbers represent time in minutes. (0.07 MB PDF) [file pbio.1000607.s005.pdf]

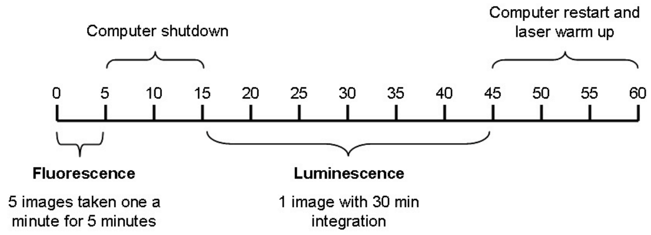

Fig. S5: Time line outlining the process of capturing sequential fluorescence and luminescence images from the same single cells. Numbers represent time in minutes.
