# Supplementary material for: Dynamic Analysis of Stochastic Transcription Cycles
Source: PLoS Biol. 2011 Apr 12;9(4):e1000607. doi: 10.1371/journal.pbio.1000607 (PMC3075210; doi:10.1371/journal.pbio.1000607)
Supplement: Figure S6 — Example plots from 17 single cells showing fluorescence (green) and luminescence (red) hPRL promoter-driven reporter construct data in unstimulated conditions over 21 h. Bottom right-hand graph shows the average fluorescence and luminescence traces from a field of cells. (0.20 MB PDF) [file pbio.1000607.s006.pdf]

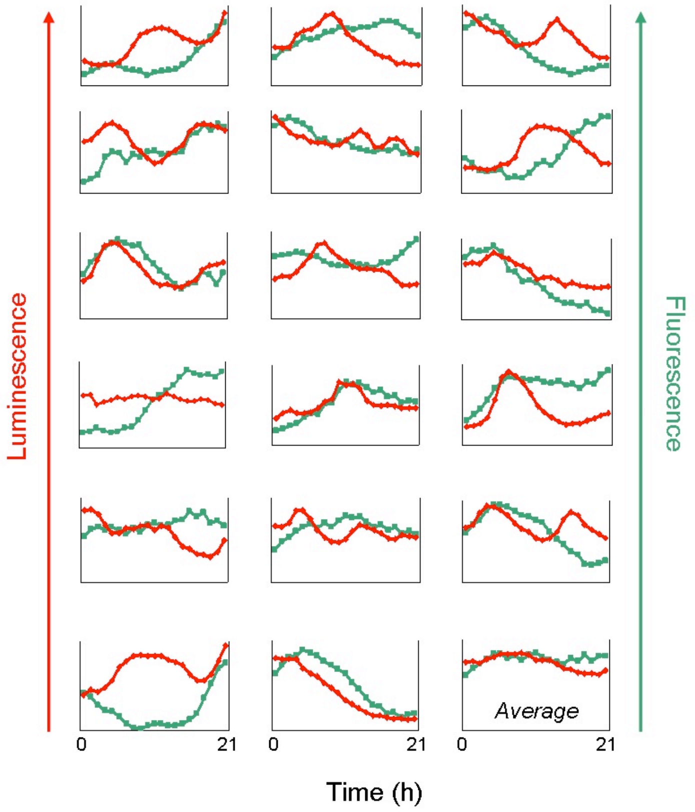

Fig. S6: Example plots from 17 single cells showing fluorescence (green) and luminescence (red) h*PRL* promoter-driven reporter construct data in unstimulated conditions over 21 hours. Bottom right-hand graph shows the average fluorescence and luminescence traces from a field of cells.
