# Supplementary material for: Dynamic Analysis of Stochastic Transcription Cycles
Source: PLoS Biol. 2011 Apr 12;9(4):e1000607. doi: 10.1371/journal.pbio.1000607 (PMC3075210; doi:10.1371/journal.pbio.1000607)
Supplement: Figure S7 — The effect of various stimuli and combinations of stimuli on expression of PRL were assessed using luminometry. (A, B) 10 ng/ml TNFa, 5 µM forskolin (FSK), and 0.5 µM BayK-8644 (BayK) were used. (C) The effect of TSA (30 ng/ml) and TSA in combination with 5 µM FSK and 0.5 µM BayK (FBK). (0.16 MB PDF) [file pbio.1000607.s007.pdf]

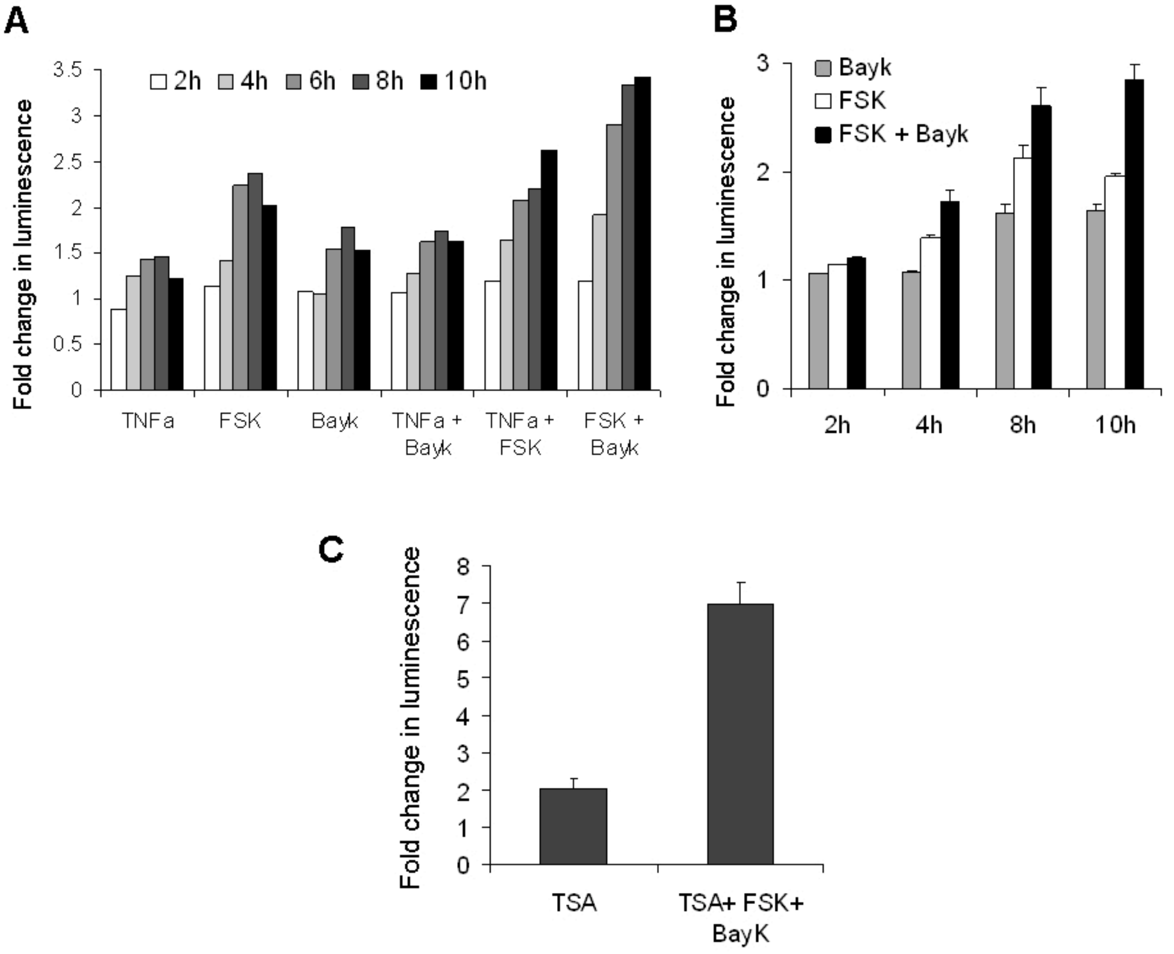

Fig. S7: The effect of various stimuli and combinations of stimuli on expression of *PRL* were assessed using luminometry. (A, B) 10ng/ml TNFa, 5μM forskolin (FSK), 0.5μM BayK-8644 (BayK) were used. (C) The effect of TSA (30ng/ml) and TSA in combination with 5μM FSK and 0.5μM BayK (FBK).
