# Supplementary material for: Dynamic Analysis of Stochastic Transcription Cycles
Source: PLoS Biol. 2011 Apr 12;9(4):e1000607. doi: 10.1371/journal.pbio.1000607 (PMC3075210; doi:10.1371/journal.pbio.1000607)
Supplement: Figure S8 — Sample ACFs of protein time series from Luc reporter constructs. Time series as in Figure 1 (B,C,E,F) of the main text. The x-axis gives the delay $s$ in hours. Each single sample ACF corresponds to the time profile observed for a single cell. Top left: Sample ACFs of time series displayed in Figure 1B of the main text (DP1, results for 15 cells, 25 hourly observations). Top right: Sample ACFs of time series displayed in Figure 1C of the main text (BAC, results for 17 cells, 95 half-hourly observations). Bottom left: Sample ACFs of time series displayed in Figure 1E of the main text (37 B, results for 24 cells, 185 half-hourly observations). Bottom right: Sample ACFs of time series displayed in Figure 1F of the main text (37 A, results for 20 cells, 95 half-hourly observations). The two bottom experiments have many cells showing longer oscillations around 25–35 h. In the experiment displayed in the bottom left graph one can see that 3 out of the 24 cells behave like a white noise process. (2.38 MB PDF) [file pbio.1000607.s008.pdf]

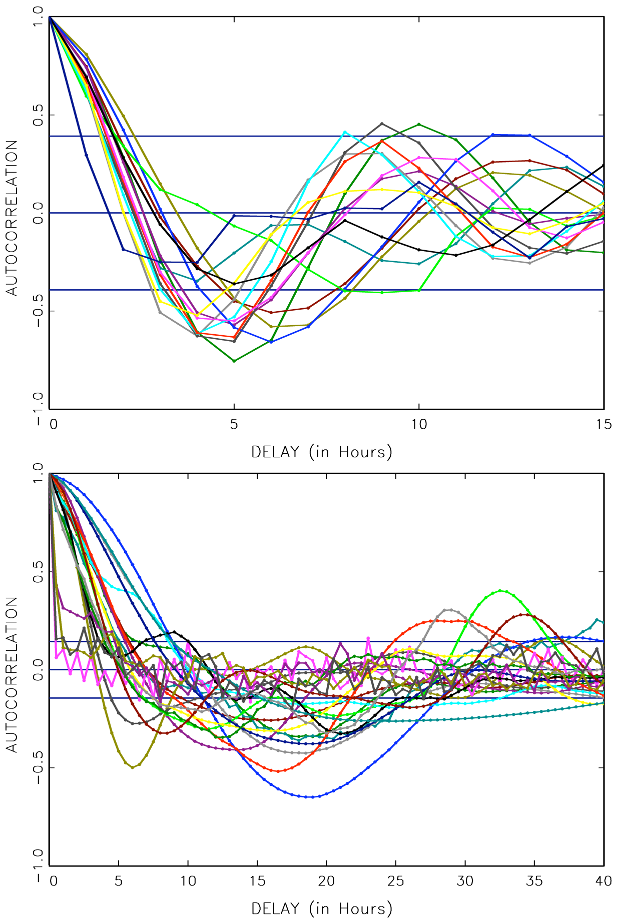

Fig. S8: Sample ACFs of protein time series from Luc reporter constructs. Time series as in Fig. 1 (B,C,E,F) of main paper. The x-axis gives the delay s in hours. Each single sample ACF corresponds to the time profile observed for a single cell. Top left: Sample ACFs of time series displayed in Fig. 1B of main paper (DP1, results for 15 cells, 25 hourly observations). Top right: Sample ACFs of time series displayed in Fig. 1C of main paper (BAC, results for 17 cells, 95 half-hourly observations). Bottom left: Sample ACFs of time series displayed in Fig. 1E of main paper (37 B, results for 24 cells, 185 half-hourly observations). Bottom right: Sample ACFs of time series displayed in Fig. 1F of main paper (37 A, results for 20 cells, 95 half-hourly observations). The two bottom experiments have many cells showing longer oscillations around 25-35 hours. In the experiment displayed in the bottom left graph one can see that 3 out of the 24 cells behave like a white noise process.
