# Supplementary material for: Dynamic Analysis of Stochastic Transcription Cycles
Source: PLoS Biol. 2011 Apr 12;9(4):e1000607. doi: 10.1371/journal.pbio.1000607 (PMC3075210; doi:10.1371/journal.pbio.1000607)
Supplement: Figure S9 — Sample ACFs of reconstructed transcription profiles from Luc (left) and d2EGFP (right) reporter constructs. The reconstruction profiles were computed using a spline approach (described below) on a fine grid of 0.1 h using the estimated posterior mean of the spline coefficients. (a) Dual experiment C1-unstim2 (21 cells, 14 h). (b) Dual experiment C1-unstim1 (29 cells, 15 h). (c) Dual experiment C2-unstim1 (21 cells, 15 h). (d) Dual experiment C2-unstim2 (15 cells, 14 h). (e) Dual experiment C1-unstim4 (20 cells, 21 h). See Table S2 for a list of dual experiments. (4.19 MB PDF) [file pbio.1000607.s009.pdf]

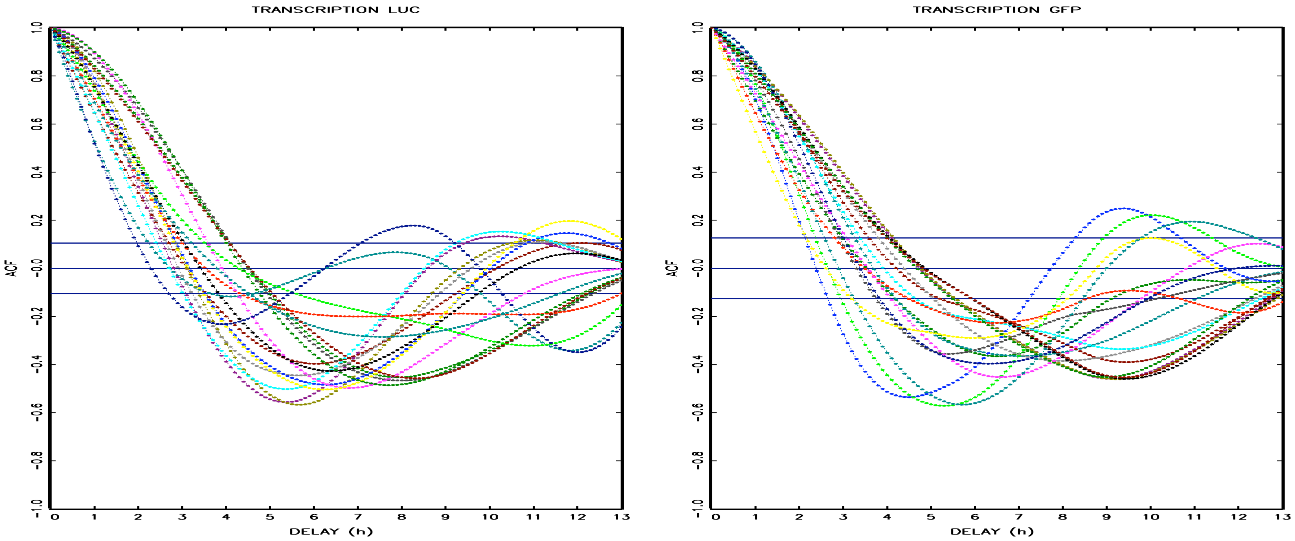

(a) Dual experiment C1-unstim2

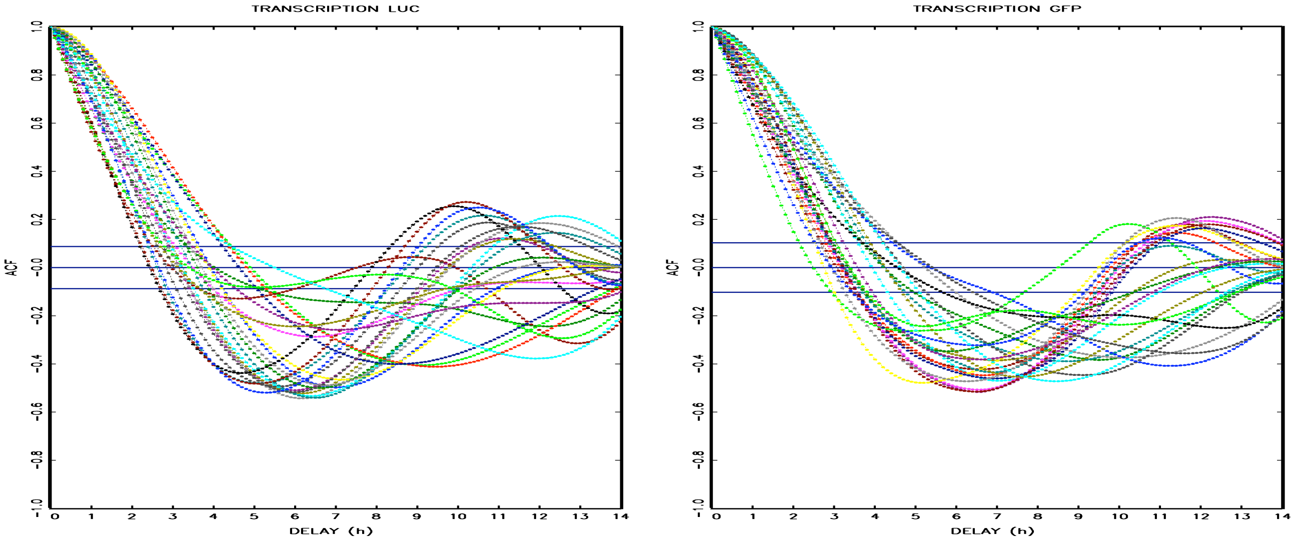

(b) Dual experiment C1-unstim1

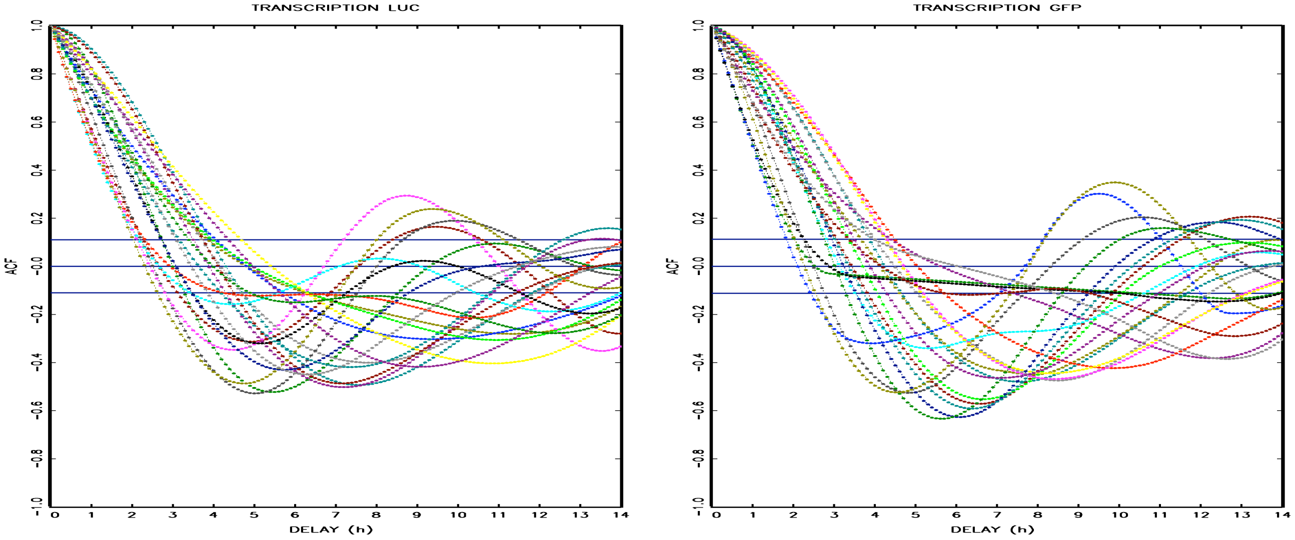

(c) Dual experiment C2-unstim1

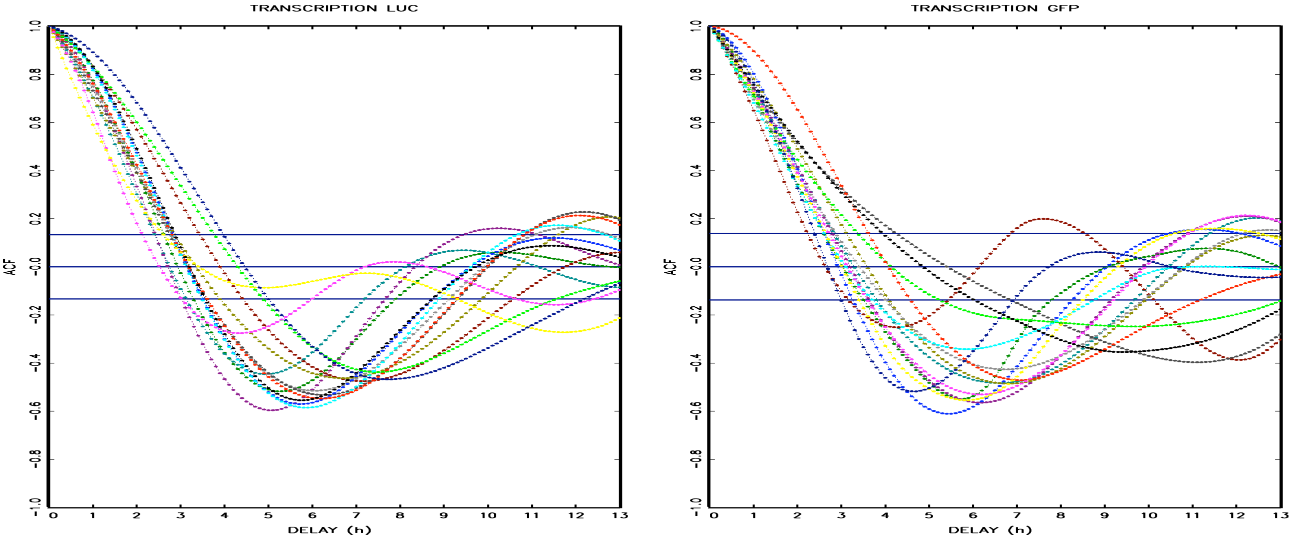

(d) Dual experiment C2-unstim2

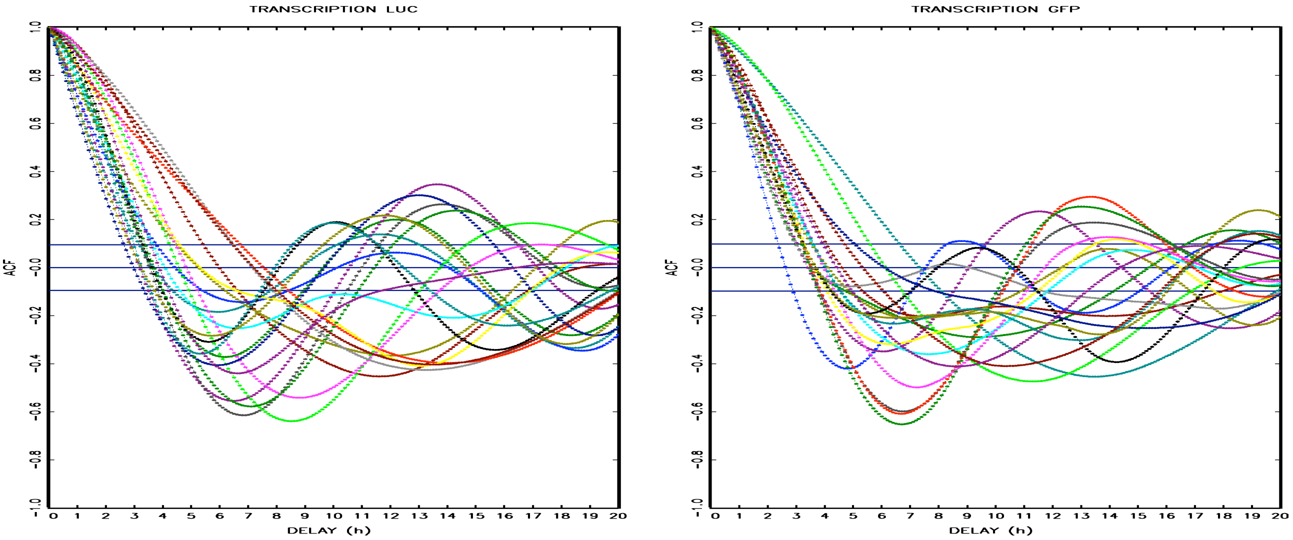

(e) Dual experiment C1-unstim4

Fig. S9: Sample ACFs of reconstructed transcription profiles from Luc (left) and d2EGFP (right) reporter constructs. The reconstruction profiles were computed using a spline approach (described below) on a fine grid of 0.1 h using the estimated posterior mean of the spline coefficients. (a) Dual experiment C1-unstim2 (21 cells, 14 hours). (b) Dual experiment C1-unstim1 (29 cells, 15 hours). (c) Dual experiment C2-unstim1 (21 cells, 15 hours). (d) Dual experiment C2-unstim2 (15 cells, 14 hours). (e) Dual experiment C1-unstim4 (20 cells, 21 hours). See Table (S2) for a list of dual experiments.
