# Supplementary material for: Dynamic Analysis of Stochastic Transcription Cycles
Source: PLoS Biol. 2011 Apr 12;9(4):e1000607. doi: 10.1371/journal.pbio.1000607 (PMC3075210; doi:10.1371/journal.pbio.1000607)
Supplement: Figure S10 — This figure shows the fit of the differential equations to the degradation data for Luc (left) and d2EGFP (right). The bottom panel shows the fit of Equation 5 to average protein data from experiments (A). The top panel shows average reconstructed mRNA profiles from protein data experiments (B) and the fit of equation (5) to the reconstructed mRNA profiles. (0.21 MB PDF) [file pbio.1000607.s010.pdf]

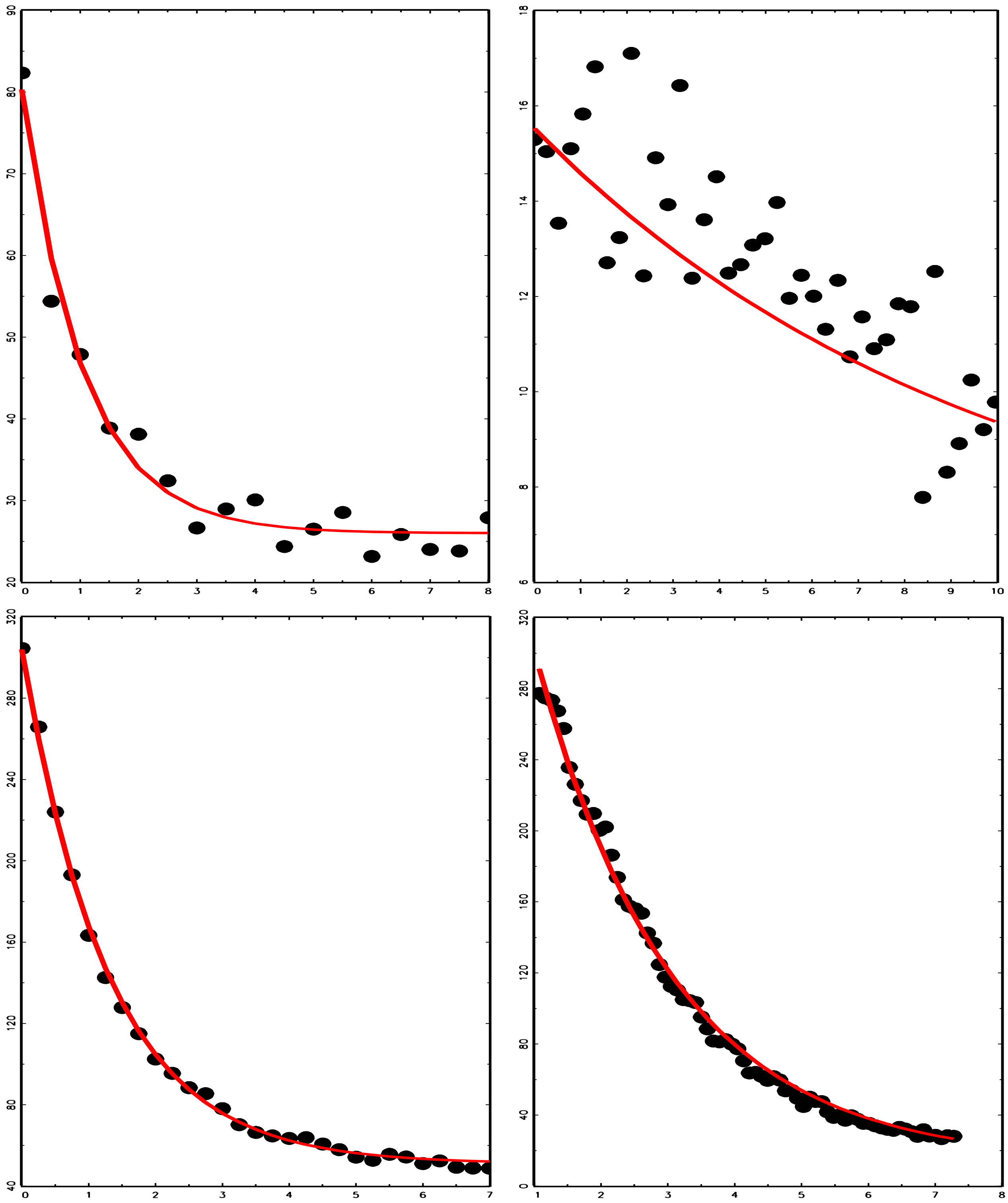

Fig. S10: This figure shows the fit of the differential equations to the degradation data for Luc (left) and d2EGFP (right). The bottom panel shows the fit of Equation (5) to average protein data from experiments (A). The top panel shows average reconstructed mRNA profiles from protein data experiments (B) and the fit of equation (6) to the reconstructed mRNA profiles.
