# Supplementary material for: Dynamic Analysis of Stochastic Transcription Cycles
Source: PLoS Biol. 2011 Apr 12;9(4):e1000607. doi: 10.1371/journal.pbio.1000607 (PMC3075210; doi:10.1371/journal.pbio.1000607)
Supplement: Figure S11 — Reconstruction of transcription profile from protein data (green, d2EGFP; red, Luc) for four randomly selected cells. The y-axis is in arbitrary units. The results for each cell are shown in a panel of three plots. Left, reconstructed transcription profile; middle, reconstructed mRNA profile; right, observed protein data (dots) together with the spline fit to the protein data. (0.30 MB PDF) [file pbio.1000607.s011.pdf]

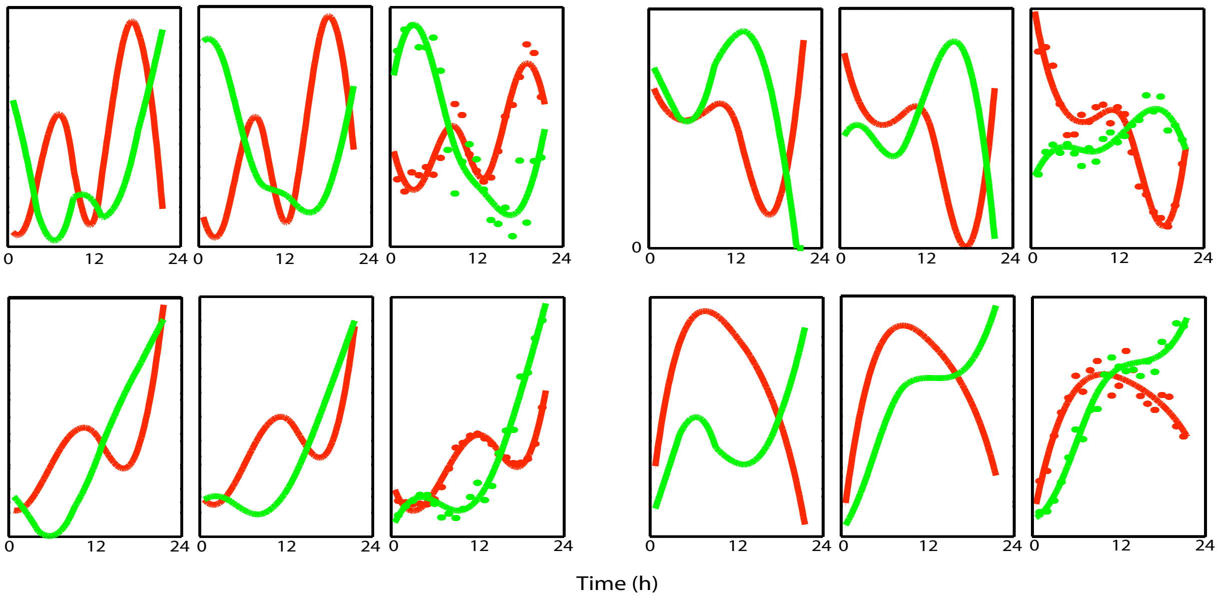

Fig. S11: Reconstruction of transcription profile from protein data (green: d2EGFP; red: Luc) for four randomly selected cells. The y-axis is in arbitrary units. The results for each cell are shown in a panel of 3 plots. Left: reconstructed transcription profile, Middle: reconstructed mRNA profile, Right: observed protein data (dots) together with the spline fit to the protein data.
