# Supplementary material for: Dynamic Analysis of Stochastic Transcription Cycles
Source: PLoS Biol. 2011 Apr 12;9(4):e1000607. doi: 10.1371/journal.pbio.1000607 (PMC3075210; doi:10.1371/journal.pbio.1000607)
Supplement: Figure S12 — Correlation plot for pooled groups: DP1 (83 cells, 3 top left panels), DP2 (36 cells, 3 top right panels), and primary (22 cells, 3 bottom panels). Each set of three panels as follows. Top panel, correlation (a) between reconstructed transcription of Luc and d2EGFP reporter; middle panel, correlation of reconstructed transcription of Luc reporter between cells within the same experiment (b); bottom panel, correlation of reconstructed transcription of d2EGFP reporter between cells within the same experiment (c). x-axis, time length over which correlation is computed; y-axis, (rank) correlation coefficient. For given time length each boxplot summarizes the distribution of the estimated correlation over the population of cells in the group by the estimated 0.025, 0.25, 0.5, 0.75, and 0.975 quantiles. The solid line gives the estimated median of each boxplot, and the dashed lines give the 95% interval for the median (points are only connected between boxplots for purpose of illustrating the trend). All top panels giving the correlation between reporter constructs are presented in the main text (confidence intervals differ slightly as they are estimated from another set of B = 4,000 bootstrap samples). (0.09 MB PDF) [file pbio.1000607.s012.pdf]

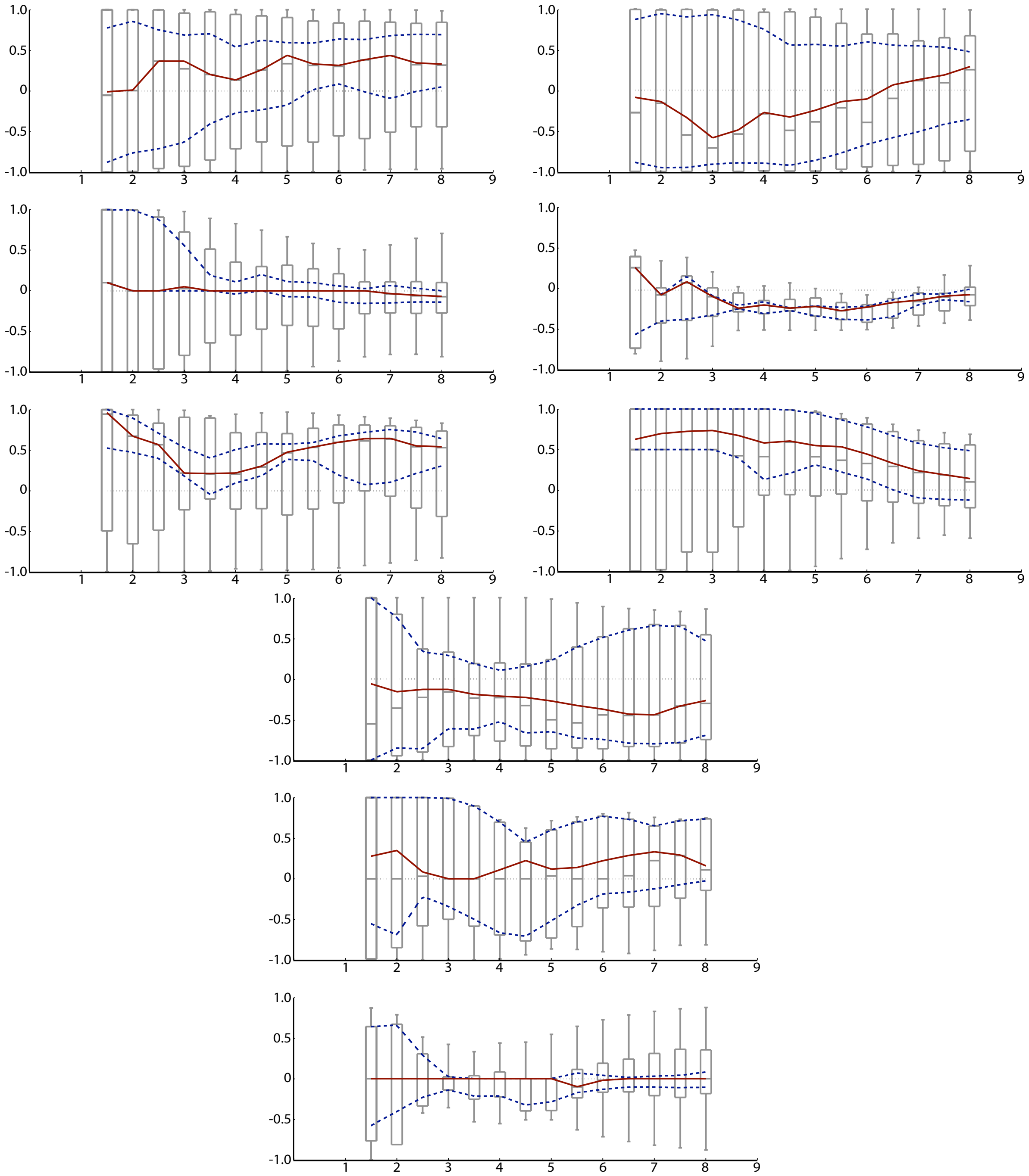

Fig. S12: Correlation plot for pooled groups: DP1 (83 cells, 3 top left panels), DP2 (36 cells, 3 top right panels), primary (22 cells, 3 bottom panels). Each set of 3 panels as follows. Top panel: correlation (a) between reconstructed transcription of Luc and d2EGFP reporter; Middle panel: correlation of reconstructed transcription of Luc reporter between cells within the same experiment (b); Bottom panel: correlation of reconstructed transcription of d2EGFP reporter between cells within the same experiment (c). X-axis: time length over which correlation is computed; Y-axis: (rank) correlation coefficient. For given time length each box-plot summarizes the distribution of the estimated correlation over the population of cells in the group by the estimated 0.025, 0.25, 0.5, 0.75, 0.975 quantiles. The solid line gives the estimated median of each box-plot, the dashed lines give the 95 % interval for the median (points are only connected between boxplots for purpose of illustrating the trend). All top panels giving the correlation between reporter constructs are presented in the main paper (confidence intervals differ slightly as they are estimated from another set of B=4000 bootstrap samples).
