# Supplementary material for: Dynamic Analysis of Stochastic Transcription Cycles
Source: PLoS Biol. 2011 Apr 12;9(4):e1000607. doi: 10.1371/journal.pbio.1000607 (PMC3075210; doi:10.1371/journal.pbio.1000607)
Supplement: Figure S13 — Correlation plot for pooled groups: unstim (119 cells, three top left panels), FBK (87 cells, three top right panels), TSA (74 cells, three bottom left panels), and TSA+FBK (41 cells, three bottom right panels). All other explanations as in Figure S12. (0.11 MB PDF) [file pbio.1000607.s013.pdf]

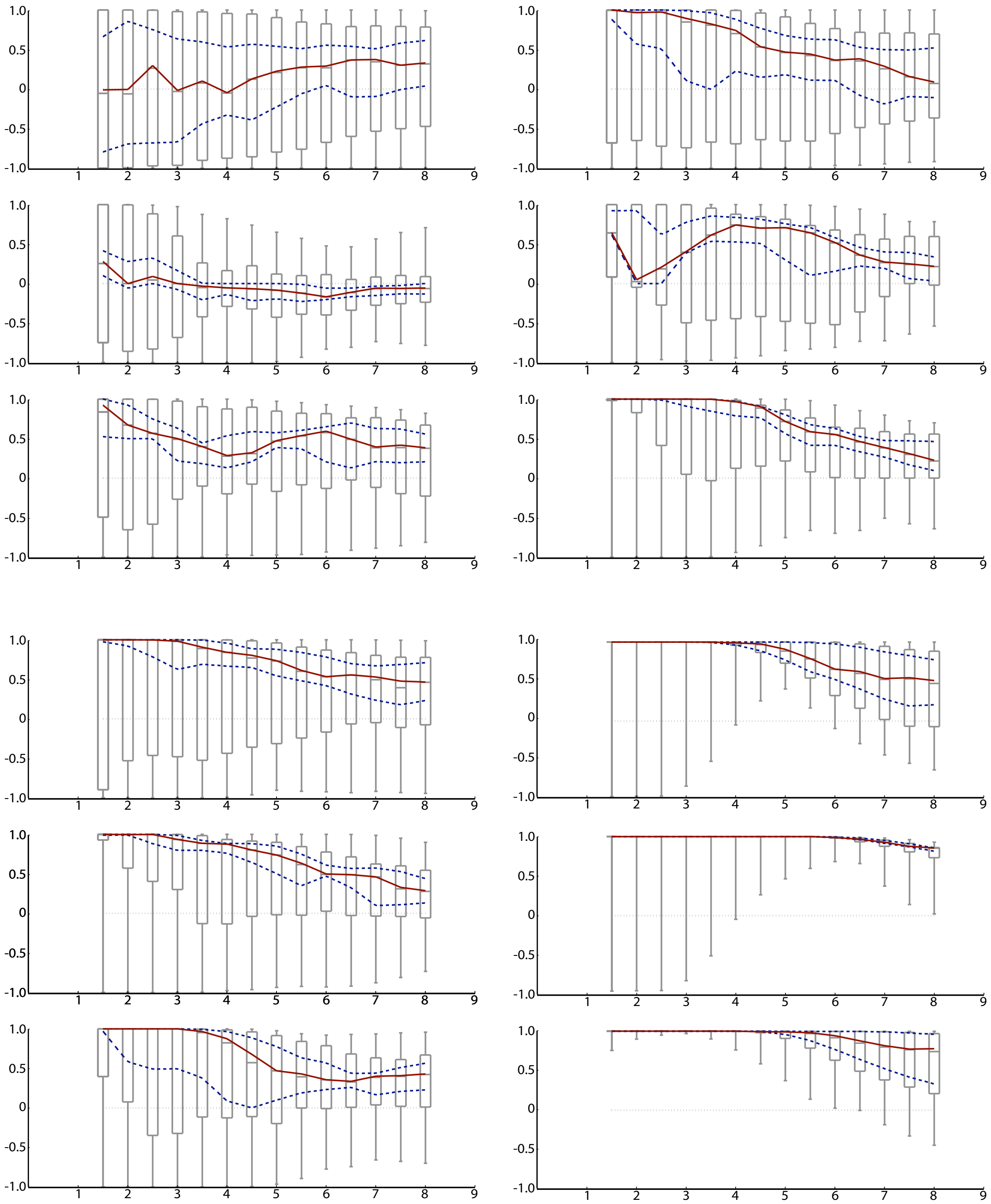

Fig. S13: Correlation plot for pooled groups: unstim (119 cells, 3 top left panels), FBK (87 cells, 3 top right panels), TSA (74 cells, 3 bottom left panels), TSA+FBK (41 cells, 3 bottom right panels). All other explanations as in Fig. (S12).
