# Supplementary material for: Dynamic Analysis of Stochastic Transcription Cycles
Source: PLoS Biol. 2011 Apr 12;9(4):e1000607. doi: 10.1371/journal.pbio.1000607 (PMC3075210; doi:10.1371/journal.pbio.1000607)
Supplement: Figure S14 — Correlation analysis for time-shifted GFP to allow for the difference d in maturation time between GFP and Luciferase. Left, d = 0.5 h. Right, d = 1 h. Row 1, TSA+FBK. Row 2, TSA. Row 3, FBK. Row 4, Unstimulated. All other explanations as in Figure S12. (0.05 MB PDF) [file pbio.1000607.s014.pdf]

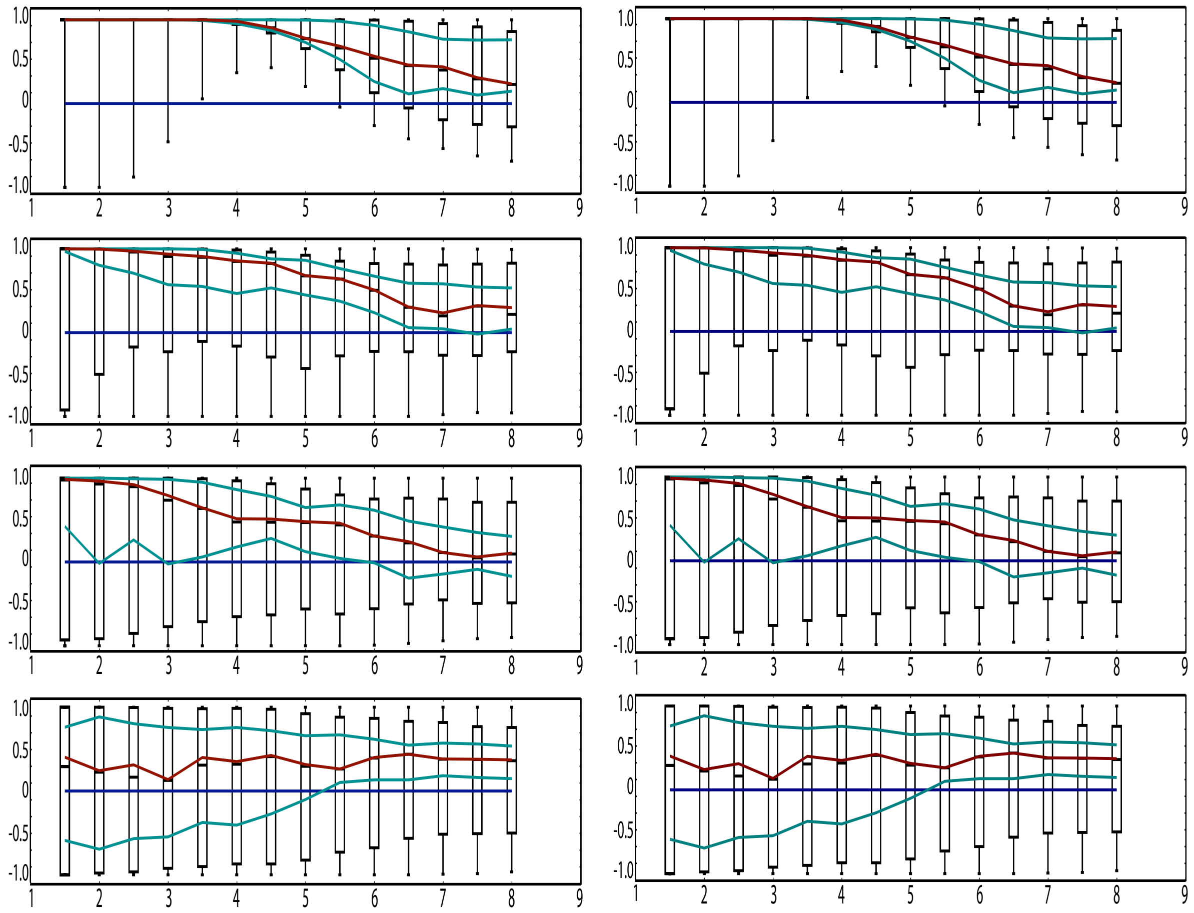

Fig. S14: Correlation analysis for time-shifted GFP to allow for the difference d in maturation time between GFP and Luciferase. Left : d= 0.5h. Right: d= 1h. Row 1: TSA + FBK. Row 2:TSA. Row 3: FBK. Row 4: Unstimulated. All other explanations as in Fig. (S12).
