# Supplementary figures and images for: Dynamic Analysis of Stochastic Transcription Cycles
Source: PLoS Biol. 2011 Apr 12;9(4):e1000607. doi: 10.1371/journal.pbio.1000607 (PMC3075210; doi:10.1371/journal.pbio.1000607)

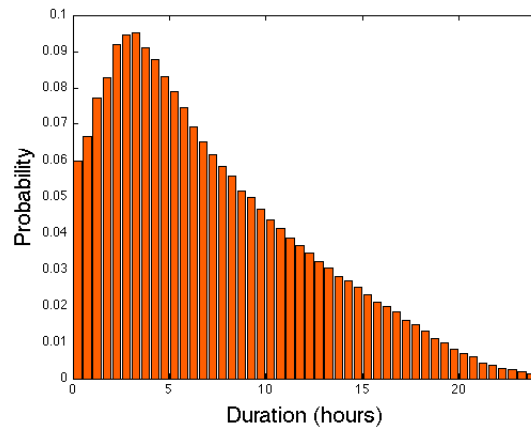

Fig. S15: The distribution of off times without weak switches removed.

Supplement: Figure S15 — The distribution of off-times without weak switches removed. (0.03 MB PDF) [file pbio.1000607.s015.pdf]
