# Supplementary material for: Dynamic Analysis of Stochastic Transcription Cycles
Source: PLoS Biol. 2011 Apr 12;9(4):e1000607. doi: 10.1371/journal.pbio.1000607 (PMC3075210; doi:10.1371/journal.pbio.1000607)
Supplement: Figure S16 — Estimated distributions of the switch times (in hours). In this case, the cell in question was determined to have three switches, and each color corresponds to the estimates for an individual switch time. (0.04 MB PDF) [file pbio.1000607.s016.pdf]

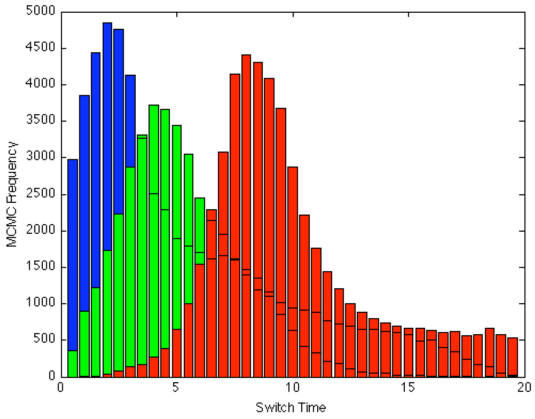

Fig. S16: Estimated distributions of the switch times (in hours). In this case, the cell in question was determined to have three switches, each colour corresponds to the estimates for an individual switch time.
