# Supplementary material for: Dynamic Analysis of Stochastic Transcription Cycles
Source: PLoS Biol. 2011 Apr 12;9(4):e1000607. doi: 10.1371/journal.pbio.1000607 (PMC3075210; doi:10.1371/journal.pbio.1000607)
Supplement: Figure S17 — The estimated distributions of , , and . (0.84 MB PDF) [file pbio.1000607.s017.pdf]

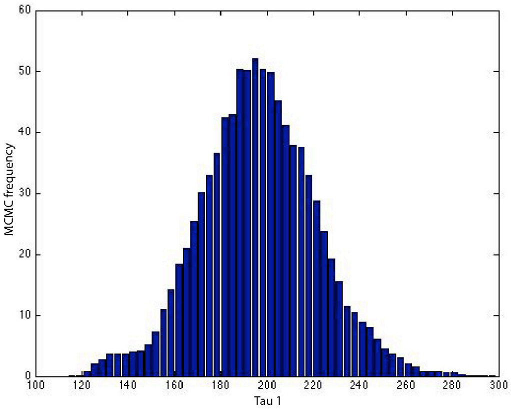

(a) Distribution of τ_1.

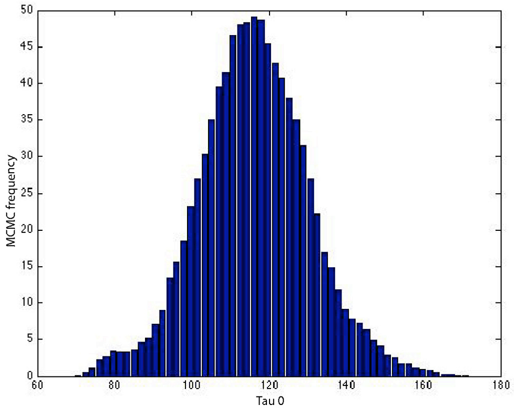

(b) Distribution of τ_0.

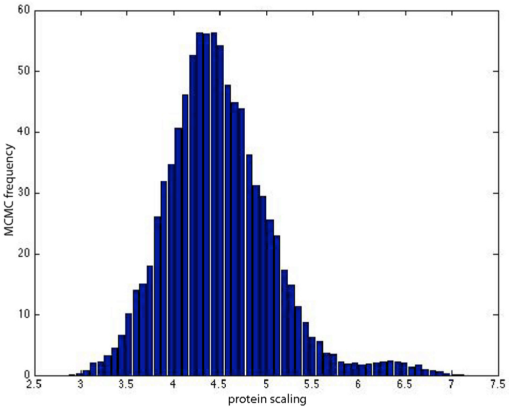

(c) Distribution of s_P.

Fig. S17: The estimated distributions of τ_1, τ_0 and s_P.
