# Supplementary material for: Dynamic Analysis of Stochastic Transcription Cycles
Source: PLoS Biol. 2011 Apr 12;9(4):e1000607. doi: 10.1371/journal.pbio.1000607 (PMC3075210; doi:10.1371/journal.pbio.1000607)
Supplement: Table S1 — Results of degradation rate estimation. Estimated and (posterior standard errors in brackets) for Luc (left) and d2EGFP (right). All rates are per hour. If data were used for more than one experiment, the average estimate is used (stated in bold) for the subsequent reconstruction of transcription and for fitting the switch model. (0.06 MB PDF) [file pbio.1000607.s018.pdf]

Experiment	δ_P (Luc)	Experiment	δ_P (d2EGFP)
Luc 1010	0.794 (0.051)	d2EGFP 0903	0.4686 (0.003)
Luc 1603	0.938 (0.057)	d2EGFP 2305	0.5126 (0.0067)
Luc 2103	0.903 (0.065)	d2EGFP 3003	0.4904 (0.0043)
Luc 1212	0.771 (0.015)		
Luc **average**	**0.8515**	**average**	**0.4905**
**half-life**	**0.815 h**	**half-life**	**1.41 h**

Experiment	δ_M (Luc)	Experiment	δ_M (d2EGFP)
Luc 0310	1.04 (0.156)	d2EGFP 0310	0.106 (0.04)
Luc 1008	0.954 (0.086)	d2EGFP 301008	0.15 (0.05)
Luc 1312	1.14 (0.18)	d2EGFP 311008	0.09 (0.02)
Luc **average**	**1.045**	**average**	**0.115**
**half-life**	**0.66 h**	**half-life**	**6 h**

Table S1: Results of degradation rate estimation. Estimated δ_P and δ_M (posterior standard errors in brackets) for Luc (left) and d2EGFP (right). All rates are per hour. If data was used for more than one experiment the average estimate is used (stated in bold) for the subsequent reconstruction of transcription and for fitting the switch model.
