# Supplementary material for: Dynamic Analysis of Stochastic Transcription Cycles
Source: PLoS Biol. 2011 Apr 12;9(4):e1000607. doi: 10.1371/journal.pbio.1000607 (PMC3075210; doi:10.1371/journal.pbio.1000607)
Supplement: Table S2 — List of dual reporter experiments. For the correlation analysis, data from unstimulated experiments were pooled according to cell type into DP1, DP2, and primary. Data were also pooled according to stimulus into unstim, FBK, TSA, and TSA+FBK. Column 4 gives time of stimulation for stimulated experiments. Column 5 gives number of cells per experiment used for analysis after discarding cells with very low amplitude (number before discarding in brackets). The final column gives number of data points measured at hourly intervals. Data for the two reporters are not taken at identical time points, but this is corrected for in the analysis. (0.03 MB PDF) [file pbio.1000607.s019.pdf]

Experiment	Cell type	Stimulus	Stimulation time	Number of Cells N	length (h)
C1-unstim1	DP1	unstim	N/A	29 (33)	15
C1-unstim2	DP1	unstim	N/A	21 (24)	14
C1-unstim3	DP1	unstim	N/A	13 (14)	15
C1-unstim4	DP1	unstim	N/A	20 (20)	21
C2-unstim1	DP2	unstim	N/A	21 (21)	15
C2-unstim2	DP2	unstim	N/A	15 (15)	14
Pr-unstim1	primary	unstim	N/A	6 (6)	20
Pr-unstim2	primary	unstim	N/A	16 (16)	19
C1-FBK1	DP1	FBK	t=1	11 (15)	13
C1-FBK2	DP1	FBK	t=1	15 (15)	17
C1-FBK3	DP1	FBK	t=1	9 (11)	12
C1-FBK4	DP1	FBK	t=4	16 (17)	13
C1-FBK5	DP1	FBK	t=1	43 (46)	10
C1-TSA1	DP1	TSA	t=7	13 (15)	17
C1-TSA2	DP1	TSA	t=4	8 (9)	14
C1-TSA3	DP1	TSA	t=4	17 (20)	14
C1-TSA4	DP1	TSA	t=1	13 (16)	16
C1-TSA5	DP1	TSA	t=1	23 (30)	9
C1-TSA-FBK1	DP1	TSA+FBK	t=2	13 (19)	15
C1-TSA-FBK2	DP1	TSA+FBK	t=2	17 (20)	15
C1-TSA-FBK3	DP1	TSA+FBK	t=1	11 (11)	9

Table S2: List of dual reporter experiments. For the correlation analysis data from unstimulated experiments were pooled according to cell type into DP1, DP2, and primary. Data were also pooled according to stimulus into unstim, FBK, TSA, and TSA+FBK. Column 4 gives time of stimulation for stimulated experiments. Column 5 gives number of cells per experiment used for analysis after discarding cells with very low amplitude (number before discarding in brackets). The final column gives number of data points measured at hourly intervals. Data for the two reporters are not taken at identical time points but this is is corrected for in the analysis.
