# Supplementary material for: Dynamic Analysis of Stochastic Transcription Cycles
Source: PLoS Biol. 2011 Apr 12;9(4):e1000607. doi: 10.1371/journal.pbio.1000607 (PMC3075210; doi:10.1371/journal.pbio.1000607)
Supplement: Text S1 — Description of experimental and theoretical methodology. (0.25 MB PDF) [file pbio.1000607.s020.pdf]

SUPPORTING INFORMATION

for

Dynamic analysis of stochastic transcription cycles

Claire V. Harper, Bärbel Finkenstädt, Dan J. Woodcock, Sönke Friedrichsen, Sabrina Semprini, Louise Ashall, David G. Spiller, John J. Mullins, David A. Rand, Julian R.E. Davis, Michael R. H. White.

Contents

1 Supplementary materials and methods **1**

2 Experimental supporting information **1**
2.1 Cell line characterization . . . 1
2.2 Choice of stimuli . . . 2

3 Theoretical supporting information **4**
3.1 Autocorrelation analysis . . . 4
3.2 Reconstruction of transcription profiles for luciferase and d2EGFP . . . 5
3.2.1 Mathematical model . . . 5
3.2.2 Estimation of degradation rates for luciferase and d2EGFP . . . 5
3.2.3 Estimation of transcription profiles . . . 6
3.3 Correlation analysis of dual reporter data . . . 7
3.4 Switch model . . . 10
3.4.1 Inference results for the switch model . . . 13
3.5 Test for memorylessness . . . 13

4 Statement of author contributions **15**

5 References **15**

1 Supplementary materials and methods

Luminometry. For luciferase assays, 10^5 cells/well were grown in 24-well plates and stimulated as indicated (0.5μM forskolin, 5μM BayK-8644). Cells were washed twice with ice cold PBS and lysates (Tris/PO4 25 mM, MgCl2 10 mM, EDTA 5 mM, 15% glycerol, 0.1% Triton X100, 0.1 mg/ml BSA) were prepared. The luciferase activity was measured using a BMG Lumistar luminometer. Three replicates were analysed for each treatment group per dish and experiments were repeated at least 2 times (shown as mean $\pm$ standard deviation).

Flow Cytometry. GH3-DP1 cells were stimulated for appropriate times, trypsinized and re-suspended in phosphate-buffered saline (PBS) before analysis by flow cytometry using a Coulter-Epics Altra flow cytometer. Wildtype GH3 cells were used to detect autofluorescence levels. 10,000 cells/sample were analyzed.

Calculation of luciferase and d2EGFP protein and mRNA degradation rates in single cells. For both luminescence and fluorescence experiments, cells were seeded into dishes as described above. Images were captured every 5 min (fluorescence) or integrated over 30 min (luminescence). Stimulus (5μM forskolin and 0.5μM BayK-8644) was added directly to the dish for 6h, followed by treatment with 10μg/ml cycloheximide (for protein degradation rate) or 3μg/ml actinomycin D (for mRNA degradation rate) and imaged for at least a further 15h. For estimation of degradation rates see supplementary Section 3.2.2.

Estimation of plasmid copy number. Genomic DNA was extracted from 5x10^4 GH3-DP1 cells (Roche) and was compared by real-time PCR to known copy numbers of h*PRL*-luciferase and h*PRL*-d2EGFP plasmid DNA using absolute quantification. Plasmid copy numbers were determined by absorption extinction co-efficients.

2 Experimental supporting information

2.1 Cell line characterization

Transcription of *PRL* has been shown to oscillate using luminescent reporter constructs (Fig. S1A-C). We show that there is an apparent cycle in the frequency of the oscillations that is not dependent on promoter length (Fig. S1D). The relative luminescence signal was however greatly affected by promoter length: the 160kbp BAC h*PRL* genomic reporter construct (comprising approximately 115kbp of $5'$-flanking sequence, intronic sequences, and 38kbp $3'$-flanking DNA) generated a 20-fold greater signal than the 5kbp promoter construct in unstimulated cells (Fig. S1D). The average cycle duration was significantly longer in primary transgenic rat pituitary cells containing the 160kbp genomic reporter construct, compared to cell lines containing either type of reporter ($p < 0.05$, t-test; Fig. S1D).

Stable cell lines were constructed with separately integrated luciferase or d2EGFP reporters under the control of the 5 kbp human *PRL* promoter into the rat pituitary cell line GH3 (GH3-DP). Two independent stably transfected cell clones were used for this study (GH3-DP1 and GH3-DP2) to exclude any possible effect of transgene integration site. The number of copies of a stably transfected reporter

construct within a single cell might affect the transcription profile obtained, so the copy number of the *PRL* promoter-reporter constructs was calculated using absolute quantification by real-time PCR. The copy number was measured to be fewer than 2 per cell for both plasmids (by comparison to the standard curve for 2 replicate experiments: Fig. S2), and therefore copy number has been considered to be 1-2 copies per cell.

Fluorescent signal from the d2EGFP construct was measured in the GH3-DP cells to assess responsiveness of the reporter over time. Fluctuations in levels of d2EGFP over short periods would not be easily detected in single cells using confocal microscopy due to the longer mRNA and protein half-lives of this reporter (see Section 3.2.2 below). Therefore flow cytometry was first used to ascertain whether the expression of this reporter was variable among single cells in unstimulated conditions. In a binary model, transcription in individual cells would be either 'on' or 'off', and proportions would differ with transcriptional activation; in a graded model, most cells would be capable of responding to the stimulus with a progressive increase in transcription, indicated schematically in green diagrams (Fig. S3, upper two panels). Typical experimental data are shown in Fig. S3, bottom panel: in the absence of stimulation, a biphasic population was detected where a subset of cells showed a high d2EGFP signal (gene expression 'on') and the remaining population were 'off' (low GFP signal). Stimulation with FBK induced an increase in the 'on' population, although the biphasic pattern of expression was maintained over long periods, suggesting a transient switching between 'on' and 'off' states over time.

The GH3-DP cell lines were imaged at a single time-point for both luminescent and fluorescent reporter constructs to enable comparison of the expression of the identical *PRL* promoters in the same single cells. Cells within the field of view were selected for dual reporter imaging using three criteria: 1) the cell must remain as a clearly discernable single cell throughout the duration of the experiment; 2) the cell must have detectable (above background) fluorescence and luminescence signal; and 3) the cell must not reach saturated fluorescence levels. Points 2 and 3 were vital criteria for using the data in the mathematical model.

To visualise fluorescent and luminescent reporter gene expression from dual constructs driven by identical *PRL* promoters within the same individual cells over time, cells were placed on a confocal microscope with a low light level CCD camera attached to the top port. Fluorescence images were captured using confocal microscopy (to reduce the background fluorescence signal from the luciferin in the culture medium). Then the microscope was shut down and a luminescence image was captured (Fig. S5). This cycle was repeated for up to 21h.

Sequential fluorescence and luminescence data were collected from single cells over time (Fig. S6). Heterogeneous expression of the two h*PRL*-driven reporter constructs was detected in unstimulated conditions. Variable response patterns were detected with cells showing no fluctuations from either reporter gene, or fluctuations from one or both reporters. The dynamics from each reporter gene were masked in the population average.

2.2 Choice of stimuli

It was important for this work to identify a stimulus that showed a rapid effect on prolactin transcription. The combined stimulus of the adenylate cyclase activator forskolin (5μM) and the L-type calcium channel activator BayK-8644 (0.5μM), a stimulus cocktail referred to as FBK, has previously been reported to elicit a synergistic increase in pituitary hormone transcription [1] which was replicated in our experimental system (Fig. S7A-B). The histone deacetylase inhibitor trichostatin A

(TSA) has previously been shown to have an effect on expression of the endogenous rat *PRL* gene in clonal rat pituitary GH4 cells [2], and was similarly found to increase expression of the *PRL*-luc reporter gene in our GH3-DP1 cells, and to increase the response to FBK (Fig. S7C).

3 Theoretical supporting information

3.1 Autocorrelation analysis

The analysis of the autocorrelation function of a time series is a commonly applied and useful descriptive tool in time series analysis (see, for example, [3] for an introduction). Let $x_1, ..., x_T$ be observations of a time series. The *sample autocovariance function* is

$$\hat{\gamma}(s) := T^{-1} \sum_{t=1}^{T-|s|} (x_{t+|s|} - \bar{x})(x_t - \bar{x}), \quad |s| < T,$$

where $\bar{x} = T^{-1} \sum_{t=1}^{T} x_t$ is the sample mean of $x_1, ..., x_T$. The sample autocovariance function thus estimates the covariance between the series and the time shifted series as a function of the length of the time shift s also called *delay*. Dividing the sample autocovariance function by the sample variance gives the *sample autocorrelation function (ACF)*

$$\hat{\rho}(s) = \frac{\hat{\gamma}(s)}{\hat{\gamma}(0)}, \quad |s| < T, \tag{1}$$

with the property $-1 \leq \hat{\rho}(s) \leq 1$. The sample autocovariance and autocorrelation function can be computed for any data set $x_1, ..., x_T$ irrespective of whether the underlying process is stationary [3]. For data containing a periodic component, the function $\hat{\rho}(s)$ will exhibit the same periodicity as the time series and thus can be used as an indicator of periodic behaviour.[1]

Fig. (S8) shows the sample ACFs for the single cell luciferase (Luc) protein data sets displayed in Fig. 1 of the main paper. The maximal value of the delay is chosen depending on sample size since the effective sample size used for the estimation of the correlation coefficient decreases with s. The sample ACFs clearly are reminiscent of oscillatory or pulsatile behaviour with a periodicity around 8 to 13 hours in the cell lines (top graphs) and longer around 25 to 30 hours in the primary transgenic cells (bottom graphs). It is obvious that the period lengths are strongly varying between the cells and also over time. The ACFs of the reconstructed transcription profiles (see below for details of the reconstruction) from the dual luciferase (Luc) and destabilized green fluorescent protein (d2EGFP) reporter constructs for five chosen dual experiments are shown in Fig. (S9). One can see that the distributions of the sample ACFs are homogeneous over the experiments and over the different reporter constructs. The behaviour is compatible with the longer Luc reporter data analysed in Fig. (S8). Fig. (S9) also shows that the reconstructed transcription profiles from the two reporter species have very similar distributions of the ACFs and thus exhibit the same type of dynamics over the populations of cells. In particular note that for any given single cell the two transcription profiles are each characterized by stochastically varying realisations of ACFs out of the distribution of all ACFs. Whilst the ACF characterizes the dynamic behaviour of the two reporters it is not clear whether their behaviour is correlated within a single cell or between cells. We focus on this question in the correlation analysis described below.

[1] Another common tool to detect periodicity is spectral analysis which is based on a frequency decomposition of the autocovariance function and the closely related spectral representation (or frequency decomposition) of the time series process itself. We found that for the applications here the spectral approach was less informative as the observational length of the time series is often only around once or twice the typical length of an oscillation.

3.2 Reconstruction of transcription profiles for luciferase and d2EGFP

3.2.1 Mathematical model

The transcriptional correlation cannot be studied directly from the imaging data because both the luciferase and the d2EGFP reporter constructs are subject to different durations associated with the underlying degradation processes. We use the following ordinary differential equations model for the reconstruction of transcription profiles from protein data (see also [4])

$$\begin{aligned} dM/dt &= \tau(t) - \delta_M M(t) \\ dP/dt &= \alpha M(t) - \delta_P P(t), \end{aligned} \tag{2}$$

where M and P denote concentration of reporter mRNA and corresponding protein, respectively. The first equation describes the dynamics of mRNA molecules with transcription function $\tau(t)$ and degradation rate δ_M. Protein is synthesized at a rate proportional to the abundance of mRNA and leaves the molecular compartment at rate δ_P. We assume that P is measured indirectly as $\tilde{P}(t) = s_P P(t)$ whilst M is unobserved. Re-parameterizing (2) in terms of $\tilde{P}$ gives a scaled model with the same functional form as (2) in which transcription becomes $\tilde{\tau}(t) = s_P \tau(t)$. To simplify notation and without loss of generality, we set $P = \tilde{P}$ and $\tau = \tilde{\tau}$ and hence (2) now denotes the scaled model. For a given solution path $P(t)$ of model (2) the unobserved mRNA profile can be solved for

$$\alpha M(t) = \frac{dP}{dt} + \delta_P P(t). \tag{3}$$

Thus the transcription profile can be reconstructed using

$$\alpha \tau(t) = \frac{d\alpha M(t)}{dt} + \delta_M \alpha M(t). \tag{4}$$

The latter is a transcription profile as it is proportional to transcription (this does not affect any correlations). We observe experimental time series at discrete time points $\mathbf{y} = \{P(t_i); i = 1, ..., T\}$. In order to study correlation at the transcriptional level the first task is to reconstruct transcription $\tau(t)$. Since M is not observed prior knowledge of the rates δ_M, δ_P facilitates identification of the transcription profiles. We first describe estimation of the rates δ_M, δ_P associated with each reporter. These will be treated as known parameters in the reconstruction of transcription profiles based on model (2).

3.2.2 Estimation of degradation rates for luciferase and d2EGFP

In order to estimate degradation rates δ_M and δ_P for both reporter constructs we used data from timecourse imaging experiments using different treatments

(A) translation of reporter protein was inhibited by adding cycloheximide (CHX) and activity of protein reporter data was sampled.

(B) transcription was inhibited by adding Actinomycin D (ActD) and the corresponding reporter protein activity was sampled.

Consider the experiment in (A): the effect of the CHX will entail no or some small translation. We therefore assume that the protein dynamics can be described by

$$dP/dt = c_P - \delta_P P(t), \tag{5}$$

where c_p is a small non-negative constant. Fitting Equation (5) to the protein reporter data from experiments in (A) thus provides an estimate of the rate δ_P. Then applying Equation (3) together with the estimated δ_P to the protein data of experiment (B) allows us to reconstruct the unobserved reporter mRNA profile. Similarly, inhibition of transcription in experiment (B) can be modeled as

$$dM/dt = c_M - \delta_M M(t), \tag{6}$$

where c_M is a small non-negative constant that is close to zero if transcription is fully inhibited. Fitting Equation (6) to the reconstructed mRNA data from experiment (B) will obtain an estimate of δ_M. For all applications here we use averages of concentrations of reporter protein over all cells in the experiment. We used a discrete Euler approximation to Equation (6) at the same grid-size as the data[2] in order to reconstruct the mRNA profiles from the average protein data of experiment (B). Equations (5) and (6) have the same functional form and were fitted using a mean ODE approach (see [4]) where the likelihood function was based on the product of independent Gamma densities which were parameterized to have a mean equal to a solution of the ODE, *i.e.* (5) or (6), and a time-constant variance.[3]

The methodology described was applied to protein data from experiments of type (A) and (B) for both reporter constructs. Table (S1) gives estimation results for degradation rates. The protein half-lives were estimated to be about 0.82 h for Luc and 1.41 h for d2EGFP, respectively, whilst the mRNA half-lives were estimated to be around 0.66 h for Luc and considerably longer around 6 h for d2EGFP. Fig. (S10) illustrates the fit of the ODEs for a selected set of experiments. Note that there is more variation observed in the fitting of Equation (6). This can be expected because the mRNA profile is computed indirectly from the protein data. The resulting signal to noise ratio is decreased because the back-calculation yields an mRNA process which is small, *i.e.* approximately at the level of growth rates of the protein, but still incorporates the noise affecting the absolute level of the observed protein. We have studied the cell-to-cell fluctuations of the decay rates for a field of cells using a Bayesian hierarchical model (unpublished manuscript) and found that there was only a very small variation between cells.

3.2.3 Estimation of transcription profiles

The rates estimated above for δ_M, δ_P associated with d2EGFP and Luc are treated as known parameters in the reconstruction based on model (2). The basic idea for our inference is to represent the solution path $P(t)$ of model (2) by a continuous function $P^*(t)$ which is fitted to the observed protein data. The transcription profile can then be reconstructed using an Euler approximation of (3) and (4) for small time intervals (here we chose length 0.1h) replacing $P(t)$ by the fitted continuous function $P^*(t)$.

For each experiment containing $i = 1, ..., N$ cells we observe protein activity for $r = 1, 2$ reporter constructs each imaged at time points $t = 1, ..., T$.[4] Thus we have a set of discrete observations

---

[2]An analysis of the degradation in the single cell data sets using a stochastic differential equations approach on a finer time grid is currently subject of our research.

[3]An analysis based on the normal distribution produced similar results. However, the gamma distribution is better suited to cope with the smaller positive signal remaining after degradation.

[4]The data for the two reporters are dual time courses. For ease of notation we use $t = 1, ..., T$ as generic index notation for the set of time points for both reporters. In practise the two reporters are not measured at identical time points. However it is straightforward to allow for this.

$\mathbf{y}_{i,r} = (y_{i,r}(1), ..., y_{i,r}(T))$. To approximate the ODE solution we use the form

$$P^*_{i,r}(t) = \mathbf{X}(t)\boldsymbol{\beta}_{i,r} \quad (7)$$

where $\mathbf{X}(t)$ is a $T \times P$ design matrix containing P spline basis functions and $\boldsymbol{\beta}_{i,r}$ contains the P spline coefficients to be estimated. Here we used simple cubic spline basis functions with 3 knots for time series with less than 14 hourly observations, 4 knots for time series with 14 to 17 observations, and 5 knots for time series with more than 17 hourly observations. The knots are spaced at approximately equal distances. The spline functions are fitted to allow for continuity at the knot points.

Assuming that the errors between the observed protein data and the spline approximation are independently and identically normally distributed in time and over cells the joint likelihood function is

$$L(\Theta_r; \mathbf{y}_r) = \prod_{i=1}^{N} \prod_{t=1}^{T} \Phi(y_{i,r}(t) - P^*_{i,r}(t); 0, \sigma_r^2), \quad (8)$$

where $\Phi(x; \mu, \sigma^2)$ denotes a normal density with mean μ and variance σ^2. The likelihood (8) is a function of the parameter vector $\Theta_r = (\boldsymbol{\beta}_r, \sigma_r^2)$ with $\boldsymbol{\beta}_r = (\boldsymbol{\beta}_{1,r}, ..., \boldsymbol{\beta}_{N,r})$ given the data $\mathbf{y}_r = (\mathbf{y}_{i,r}; i = 1, ..., N)$. By Bayes' theorem the posterior distribution is

$$f(\Theta_r|\mathbf{y}_r) \propto L(\Theta_r; \mathbf{y}_r)\pi(\Theta_r),$$

where L is the likelihood function (8) and $\pi(\Theta_r)$ are prior distributions which reflect our prior knowledge about Θ_r. We chose all prior distributions to be uninformative. Sampling from the posterior is achieved using a Markov chain Monte Carlo (MCMC) algorithm. The full algorithm was applied separately to all experiments with dual reporter data (list of experiments in Table S2). As a result of this estimation we obtain posterior estimates of the spline coefficients $\boldsymbol{\beta}_{i,r}; r = 1, 2; i = 1, ..., N$ which allow us to evaluate $P^*_{i,r}(t)$ at any fine grid-size and reconstruct the underlying transcription profile applying a discrete Euler approximation to the equations in (3) and (4) using the assumed degradation rates for δ_M and δ_P. Fig. (S11) demonstrates the reconstruction of the transcription profiles from the protein data using the posterior mean estimates of the spline coefficients. Before fitting the model all dual reporter time series data was screened and all cells with an extremely small amplitude in the dynamics of the measured protein in comparison to other cells of the same experiment and same reporter were discarded (this procedure resulted in a small number of cells per experiment to be discarded, see Table S2). Each time series was scaled by its mean to give concentrations relative to the mean. The resulting time series are thus centered around one.

3.3 Correlation analysis of dual reporter data

The aim is to study the correlation in transcription of the dual reporter constructs within a cell and also between the cells in any given experiment. We address this by computing rank correlation coefficients.[5] Using the posterior mean estimates of the spline coefficients we reconstruct the transcription profile of the two reporter species. For each cell we compute the rank correlation coefficient between

[5]The ordinary (Pearson) correlation and the rank correlation coefficients give identical results if the relationship between two continuous variables is linear. Here we chose to use the rank correlation as it will indicate any monotonic relationship between a pair of variables and thus is more robust to nonlinearities. We did investigate the Pearson correlation as well but did not find notably different results.

the two reconstructed transcription profiles of the two reporters d2EGFP and Luc within a cell (correlation A) and also the average rank correlation coefficient of the reconstructed profile of a given cell with all other cells in the same experiment for the Luc reporter (correlation B) and the d2EGFP reporter (correlation C). Thus, correlation A verifies whether the transcriptional pulses within a cell are in phase with each other while correlation B,C gives information about whether the transcriptional dynamics are in phase between the cells. As this may vary over time in particular for stimulated experiments all correlations are computed as a function of the length of time since a stimulus (TSA, FBK) was added starting from 1.5h (to allow for a reasonable minimal length over which any correlation is computed) to 8h. For unstimulated experiments we computed correlations after 2h into the experiment to avoid any initial bias. All experimental data were pooled into groups (see Table S2) according to cell type (DP1, DP2, primary) and stimulus (unstim, FBK, TSA, TSA+FBK) and the correlation results are computed per group. The results are plotted in figure (S12) according to cell type and in figure (S13) according to stimulus group. The sequence of box-plots summarizes the distribution of the correlation over the cells in the group against the time length over which the correlation was computed. These distributions may be asymmetric and therefore we use the median as a measure of central location. To test the hypothesis whether the median is significantly different from zero, we provide 95% confidence intervals for the median at each time length. These are obtained from drawing B (here chosen to be 4000) samples of size equal to the number of cells in the group from the set of estimated correlation coefficients and re-estimating the median. This gives a simulated distribution of the median and a 95% confidence interval can be estimated by the 0.025 and 0.975 quantiles. These are plotted together with the median in all graphs. If a confidence interval includes zero then we cannot conclude that the centre of the distribution of the correlations over the population of cells is different from zero.

The results of the correlation analysis between the two different reporter constructs are summarized as follows. Generally, it can be noted that in unstimulated conditions no significant correlation pattern can be found between the timing of expression of the two reporter genes. The distribution of the correlation coefficient has a very wide interquartile range (that is the central 50% range given by the box in a boxplot) indicating that we observe a large variation between the cells ranging from large negative to large positive correlation. The confidence intervals for the median are generally wide for all unstimulated groups (unstim, DP1, DP2, primary) and well compatible with the null hypothesis that on average the expression of the reporters within cells are uncorrelated. The reader might notice that for the unstim group (which includes the DP1 group) the median is slightly more positive for increased time length. However we cannot conclude that this is significant as the lower values of the confidence intervals are still close to or smaller than zero.

Correlations are considerably higher and a pattern appears when we consider stimulated conditions (FBK, TSA, TSA+FBK). The highest correlation results are found for group TSA+FBK. Here we observe a median correlation of one (with the interquartile range comprising values close to one) for up to 4h time length. The reconstructed transcription profiles show that almost all cells start transcribing both reporters' mRNA straight after TSA+FBK were added. Although the median correlation declines after 4h it is still significantly positive for up to 8h (which was the maximal length over which we estimated correlation). The decline itself is a consequence of the fact that there are slight differences between the cells in the lengths of their active phases. However, transcription jointly declines for both reporters at similar times in the early second half of the experiment which causes an overall significant positive correlation also on the inactive phases generally appearing after 4h.

The same pattern is observed for the TSA group with only marginally weaker correlations (here

the median stays around one for up to 3h, then declines but remaining significantly positive). It is also discernible for the FBK group although with noticeably smaller correlations (the median correlation is around 1 for a length of up to 2.5 h, then declines and is not significantly different from zero after about 6h). In the latter group a larger proportion of cells than in the TSA treated groups does not start transcribing both reporters' mRNA jointly after FBK is added. This happens in particular if one reporter appears to be in the peak or downswing of a cycle and does not seem to be able to alter its dynamic behaviour when the stimulus is added. The ranking in correlation results from FBK (weakest) to TSA and TSA+FBK (strongest) is in perfect agreement with the results of the switch model estimation of some longer Luc time series from the three stimulated groups (displayed in Fig. 8 C,D,E of main paper). Figs (S12),(S13) also show the correlations between-cells for Luc and d2EGFP for all groups. They are generally in line with the between-reporter within-cell correlation results. However, for the d2EGFP it is apparent that in some experiments the protein time series have positively correlated measurement errors between the cells. This is the reason for the correlation between d2EGFP reporters between-cells to appear positive in some unstimulated groups (unstim, DP1, DP2). The correlation patterns for the between-cell Luc reporter transcription is generally similar to the within-cell between-reporter correlation patterns described above.

The question of estimating and including protein maturation times is addressed in [5]. In calculating the correlations, the relevant quantity is the difference in maturation times between the two reporters. We have therefore included this process with a constant difference of up to 1h. We have verified that such delays do not change our correlation results. This can be seen from the following Fig.(S14) which shows between-reporter correlations obtained with 0.5h and 1h difference. It is clear from this that if we instead assumed an exponentially distributed delay with a similar difference in means then this would not affect the correlation results.

3.4 Switch model

The aim of this modeling approach is to use some of the longer and more frequently measured Luc reporter single cell time series imaging data and to specifically estimate the transcriptional on and off-times by fitting a stochastic switch model to this data. By assuming that rates are event probabilities the ODE model in Equation (2) can be transformed into the corresponding stochastic differential equations (SDE) model that explicitly incorporates the stochasticity inherent in the process [6, 7]

$$dM = \zeta_M(t)dt + \sigma_M(t)dW_M, \tag{9}$$
$$dP = \zeta_P(t)dt + \sigma_P(t)dW_P, \tag{10}$$

where W_M and W_P are independent Wiener processes,

$$\zeta_M(t) = \tau(t) - \delta_m M(t), \quad \zeta_P(t) = \alpha M(t) - \delta_P P(t) \tag{11}$$

are known as the *drift* functions, and the *volatility* functions are

$$\sigma_M(t) = \sqrt{\tau(t) + \delta_m M(t)} \quad \sigma_P(t) = \sqrt{\alpha M(t) + \delta_P P(t))}. \tag{12}$$

Notice that the drift functions are equivalent to the ODE model (2). As before, we incorporate the indirect nature of the measurement by including a scaling factor so that $\tilde{P}(t) = \frac{P(t)}{s_p}$. Substituting this into the protein Equation yields

$$\begin{aligned} \zeta_{\tilde{P}}(t) &= s_P \alpha M(t) - \delta_P \tilde{P}(t), \\ \sigma_{\tilde{P}}(t) &= \sqrt{s_P}\sqrt{s_P \alpha M(t) + \delta_P \tilde{P}(t)}, \end{aligned} \tag{13}$$

so the scaling factor will have to be inferred along with the rest of the parameters. As before, we shall henceforth only refer to the scaled protein equations and so will drop the $\sim$ notation and refer to the scaled Equation using $P(t)$.

For the switch model we use a simple function where τ can only assume two constant values corresponding to an *on-phase* of mRNA transcription, where the transcription activity is high, and an *off-phase*, where the transcription activity is low, *i.e.*

$$\tau(t) = \begin{cases} \tau_1 & \text{if } t \text{ is during an on-phase} \\ \tau_0 & \text{if } t \text{ is during an off-phase} \end{cases}$$

We call the point where τ changes a *switch* and refer to the time period from one switch to the next as a *regime*. Estimation of the switch times and therefore of the length of the regimes (except for the regimes at the boundaries of the series) is of great importance. The strength of the switch model lies in its simplicity due to a parsimonious parameterization (it only requires three parameters: two values of τ that correspond to the active and inactive phases and a vector of values to store the times when the switches occur) and interpretability. Parameter inference for the switch model is achieved using a MCMC algorithm. The likelihood function is derived from the sequence of transition densities $p(Y(s)|Y(t), \Theta)$, that is the probability of the value of the data point $Y(s)$ given the value of the previous data point $Y(t)$ evaluated for the sequence of discrete data points under the law of the SDE with parameter vector Θ. Note that an SDE of the form

$$dY(t) = \kappa(\theta - Y(t))dt + \sigma\sqrt{Y(t)}dW(t), \tag{14}$$

is known as the Cox-Ingersoll-Ross (CIR) model [8] for which there exists a closed form solution for the resulting transition densities and thus for the likelihood function. Our model equations can be changed into this form with a simple transformation and so we incorporate this solution into our likelihood function. The transition density solution to Equation (14) is

$$p(Y(s)|Y(t)) = ce^{-u-v}\left(\frac{v}{u}\right)^{q/2} I_q(2(uv)^{1/2}),$$

where

$$\begin{aligned} c &= \frac{2\kappa}{\sigma^2(1-e^{-\kappa(s-t)})}, && (15)\\ u &= cY(t)e^{-\kappa(s-t)}, && (16)\\ v &= cY(s), && (17)\\ q &= \frac{2\kappa\theta}{\sigma^2} - 1, && (18) \end{aligned}$$

and $I_q(\cdot)$ is the modified Bessel function of the first kind with fractional order q. In using this as a likelihood function for the SDE system (9)- (10), we need to be aware that it is only an exact solution when the parameters a, b and c in Equation (14) are fixed through the interval $[t, s]$. This is pertinent to the protein equation (10) as $M(t)$ is a function of time and therefore is not constrained to remain fixed throughout the interval. In our likelihood function, we circumvent this problem by using the integral of M over the interval $\hat{M}(t) = \int_t^s M(t)dt$ as an approximation. We need to calculate likelihoods for both equations of the system. As M is unobserved we need to create and update a latent time series. This requires that we create a likelihood for the mRNA series as well as the data series. The transition density corresponding to Equation (9) is given by

$$p(M(s)|M(t),\Theta) = c_M e^{-u_M-v_M}\left(\frac{v_M}{u_M}\right)^{q_M/2} I_{q_M}(2(u_M v_M)^{1/2}),$$

where

$$\begin{aligned} c_M &= \frac{2}{\delta_M(1-e^{-\delta_M(s-t)})},\\ u_M &= c_M(\tau(t)+\delta_M M(t))e^{-\delta_M(s-t)},\\ v_M &= c_M(\tau(s)+\delta_M M(s)),\\ q_M &= \frac{4\tau(t)}{\delta_M} - 1. \end{aligned}$$

The transition density corresponding to Equation (10) is

$$p(P(s)|P(t),\Theta) = c_P e^{-u_P-v_P}\left(\frac{v_P}{u_P}\right)^{q_P/2} I_{q_P}(2(u_P v_P)^{1/2}),$$

where

$$
\begin{aligned}
c_P &= \frac{2}{s_P\delta_P(1-e^{-\delta_P(s-t)})},\\
u_P &= c_P(s_P\alpha\hat{M}(t)+\delta_P P(t))-e^{-\delta_P(s-t)},\\
v_P &= c_P(s_P\alpha\hat{M}(t)+\delta_P P(s)),\\
q_P &= \frac{4\alpha\hat{M}(t)}{\delta_P}-1.
\end{aligned}
$$

The likelihoods are given by

$$
L(\Theta, M) = \prod_{i=0}^{T-1} p(M(i+1)|M(i),\Theta), \tag{19}
$$

$$
L(\Theta, P) = \prod_{j=0}^{T-1} p(P(j+1)|P(j),\Theta), \tag{20}
$$

and we are assuming uniform priors. These can then be used for the updating of parameters using MCMC. The degradation rates are presumed known (from supplementary Section 3.2.2), meaning the only other significant difference is the inference of the switch times. The main difficulty is that, as the number of switches was unknown, we had to employ a variation of the reversible jump method [9] as we effectively alter the size of the parameter space when we add or remove switches. Due to the nature of the data set however, it was necessary to enforce a set of 'moves' as the change in parameter space cannot be effected from a distribution. So instead, each move was assigned a mutually exclusive probability of 0.1 of being proposed. The possible moves are:

1. a single switch is added at random at any point in the time period of the data set,
2. a single switch is removed at random from the switch vector,
3. two switches are added an hour apart at random at any point in the time period of the data set,
4. the switch vector is searched and two consecutive switches are removed subject to the condition that they are within 15 minutes of each other. If there are more than one pair of switches that are within 15 minutes of each other, one pair is picked at random.

The likelihoods before and after these moves are compared and a proposed move is accepted or rejected in the usual manner. We incorporated this in a two stage estimation process whereby the full parameter set including the number of switches was estimated in the first stage. The value for the number of switches was then fixed with the modal value (after a burn in period) from stage one and the rest of the parameter set was estimated in the second stage. We found that this approach facilitated the convergence of the Markov chain of estimates.

Sometimes the algorithm would assign two switches very close together which would have a minimal effect on the likelihood and therefore be accepted. To remove the influence of these 'weak switches' and therefore give a more accurate representation of the duration estimates we simply discounted any off duration that led to a change in the protein level that was less than some threshold value ε multiplied by the maximum value. In practice, ε was set very low at 0.05 so as not to adversely affect the position of the distribution mode. The equivalent distribution to that in Figure 9B without this threshold value is shown in Fig. S15 for comparison.

3.4.1 Inference results for the switch model

Fig. (S17) shows the estimated posterior distributions of τ_1, τ_0 and s_P. These indicate that the algorithm is working correctly and that it has converged to a suitable value for each parameter. We take the mode of these distributions to be our estimate of the value in the mRNA reconstruction as shown for a selected cell in Fig. 5A of main paper. Fig. (S16) shows the estimated distributions of the switch times for the representative cell. Each different colour represents a separate switch time. These distributions are not as smooth as the other parameter estimates due to the discrete nature of the recording. They do, however, show a peak corresponding to the most likely value so we again take the mode of each distribution to be our estimate of the corresponding switch time.

In Fig. 5 of the main paper, the duration of the an 'on' regime was calculated by subtracting the switch time corresponding to an 'on' switch from the switch time of the subsequent 'off' switch. The duration of an 'off' regime was determined in a similar fashion. The duration of the period of the oscillation was taken to be the time from one 'on' switch to the next and calculated by subtracting the time of the second 'on' switch from the first.

In Fig. 8B of the main paper, the percentage of oscillating cells is ascertained by calculating, for each stimulus, the percentage of cells in the sample with three or more switches. For Fig. 8D of main paper, the 'time to the first active phase' is the time taken from the start of the recording to the first 'on' switch. This corresponds to the time to the first 'on' switch from when the stimulus was added for the FBK, TSA and TSA+FBK experiments, and can be interpreted as the duration to the first 'on' switch from a random time point in the unstimulated cells. The 'duration of active phase' results displayed in Fig. 8E of the main paper are calculated in the same way as the duration of an 'on' regime above, except that in the event of two or more active phases it is only the first 'on' regime of each cell that is used. The transcription rates in Fig. 4F of main paper are the results obtained from the values of τ_1 estimated from the individual cells for each stimulus.

3.5 Test for memorylessness

The probability of waiting more than t minutes until the next switch given a previous wait of s hours is given as $P(T > s + t | T > s)$ where T is the time of the next switch and $s, t > 0$. We can write this as

$$P(T > s + t | T > s) = \frac{P(T > s + t \cap T > s)}{P(T > s)}, \tag{21}$$

Now, as $P(T > s + t \cap T > s) = P(T > s + t)$ when $t > 0$, Equation 21 becomes

$$P(T > s + t | T > s) = \frac{P(T > s + t)}{P(T > s)}. \tag{22}$$

This is equal to

$$\frac{1 - P(T < s + t)}{1 - P(T < s)} = \frac{1 - F(s + t)}{1 - F(s)}, \tag{23}$$

where $F(\cdot)$ is the cumulative distribution function. For an exponential distribution,

$$F(x, \lambda) = 1 - e^{-\lambda x}, \tag{24}$$

and so we can write

$$\frac{P(T > s + t)}{P(T > s)} = \frac{e^{-\lambda(s+t)}}{e^{-\lambda s}} = e^{-\lambda t}. \tag{25}$$

As $e^{-\lambda t} = P(T > t)$, is not a function of s, we conclude that the probability of waiting an additional t hours until the next event is independent of the time already waited s.

To ascertain if our estimated off duration distributions are memoryless, we plot $P(T > s+t|T > s)$ against s for a number of values of $t = 0, 0.5, 1, \ldots, 20$. $P(T > s+t|T > s)$ is calculated as in Equation 23 with a discrete estimate of the cumulative distribution function calculated by binning the data at intervals of 0.1. By plotting these alongside the equivalent values in an exponential distribution (Fig. 9 of the main paper), we are able to determine if the distribution of off durations has the memoryless property.

4 Statement of author contributions

Claire Harper planned and performed the experiments and image analysis, prepared the figures and wrote the manuscript. Bärbel Finkenstädt developed the mathematical models, performed mathematical analysis of the imaging data, contributed to many of the figures and to preparation of the manuscript. Dan Woodcock developed and applied the binary switch model and contributed key figures and assisted with the manuscript. Sönke Friedrichsen prepared the cell lines for the work and assisted in the initial planning of experiments. Sabrina Semprini generated the BAC constructs and the transgenic rats. Louise Ashall assisted with Chromatin Immunoprecipitation assays. Dave Spiller directed the Centre for Cell Imaging, provided imaging support and commented on the manuscript. John Mullins directed the BAC construct work, generation of the transgenic rats and commented on the manuscript. David Rand planned and led the mathematical modelling and contributed key sections of the manuscript. Julian Davis directed the project and contributed to the manuscript. Michael White directed the project, supervised the experiments and wrote the manuscript.

5 References

[1] M. Szabo, N, E. Staib, B, Collins, J, & Cuttler, L. (1990) Biphasic action of forskolin on growth hormone and prolactin secretion by rat anterior pituitary cells in vitro. *Endocrinology* **127**, 1811–1817.

[2] Liu, J. C, Baker, R. E, W. Chow, C. K. S, & Elsholtz, H. P. (2005) Epigenetic mechanisms in the dopamine d2 receptor-dependent inhibition of the prolactin gene. *Molecular Endocrinology* **19**, 1904–1917.

[3] Brockwell, P. J & Davis, R. A. (2002) *Introduction to Time Series and Forecasting, 2nd ed.* (Springer-Verlag, New York).

[4] Finkenstädt, B. F, Heron, E. A, Komorowski, M, Edwards, K, Tang, S, Harper, C. V, Davis, J. R. E, White, M. R. H, Millar, A. J, & Rand, D. A. (2008) Reconstruction of transcriptional dynamics from gene reporter data using differential equations. *Bioinformatics* **24**, 2901 – 2907.

[5] Komorowski, M, Finkenstädt, B, & Rand, D. A. (2010) Using single fluorescent reporter gene to infer half-life of extrinsic noise and other parameters of gene expression. *Biophysical Journal* **98**, 2759–2769.

[6] Allen, J. S. (2003) *An introduction to stochastic processes with applications in Biology*. (Pearson Prentice Hall, New Jersey).

[7] Heron, E. A, Finkenstädt, B. F, & Rand, D. A. (2007) Bayesian inference for dynamic transcriptional regulation; the hes1 system as a case study. *Bioinformatics* **23**, 2589–2595.

[8] Cox, J. C, Ingersoll, J. E, & Ross, S. A. (1985) A theory of the term structure of interest rates. *Econometrica* **53**, 385–407.

[9] Green, P. J. (1995) Reversible jump markov chain monte carlo computation and bayesian model determination. *Biometrika* **84**, 711–732.
